# Gelsolin Contributes to the Motility of A375 Melanoma Cells and This Activity Is Mediated by the Fibrous Extracellular Matrix Protein Profile

**DOI:** 10.3390/cells10081848

**Published:** 2021-07-21

**Authors:** Ewa Mazurkiewicz, Aleksandra Makowiecka, Ewa Mrówczyńska, Iryna Kopernyk, Dorota Nowak, Antonina Joanna Mazur

**Affiliations:** Department of Cell Pathology, Faculty of Biotechnology, University of Wroclaw, 50-383 Wroclaw, Poland; ewa.mazurkiewicz@uwr.edu.pl (E.M.); a.makowiecka1@gmail.com (A.M.); ewa.mrowczynska@uwr.edu.pl (E.M.); iryna.kopernyk@uwr.edu.pl (I.K.); dorota.nowak@uwr.edu.pl (D.N.)

**Keywords:** gelsolin (GSN), CRISPR/Cas9(D10A) technique, melanoma, motility, invasion, extracellular matrix (ECM), laminin, fibronectin, collagen, Matrigel, actin cytoskeleton, SIM

## Abstract

Skin melanocytes reside on the basement membrane (BM), which is mainly composed of laminin, collagen type IV, and proteoglycans. For melanoma cells, in order to invade into the skin, melanocytes must cross the BM. It has been reported that changes in the composition of the BM accompany melanocytes tumorigenesis. Previously, we reported high gelsolin (GSN)—an actin-binding protein—levels in melanoma cell lines and GSN’s importance for migration of A375 cells. Here we investigate whether melanoma cells migrate differently depending on the type of fibrous extracellular matrix protein. We obtained A375 melanoma cells deprived of GSN synthesis and tested their migratory properties on laminin, collagens type I and IV, fibronectin, and Matrigel, which resembles the skin’s BM. We applied confocal and structured illuminated microscopy (SIM), gelatin degradation, and diverse motility assays to assess GSN’s influence on parameters associated with cells’ ability to protrude. We show that GSN is important for melanoma cell migration, predominantly on laminin, which is one of the main components of the skin’s BM.

## 1. Introduction

Gelsolin (GSN) is an actin-binding protein [1,2,3] with a controversial role in tumorigenesis. On the one hand, it seems that GSN is pro-tumorigenic, and on the other hand, there are studies showing that this protein is a tumor suppressor (discussed elsewhere [4,5]). There are only a few papers focusing on GSN’s role in the biology of melanoma. In one of our previous publications, we show that silencing of *GSN* expression with siRNA led to changes in A375 cells’ morphology, resulting in diminished cells motility [6]. However, that study was limited to the evaluation of actin polymerization state and ability to cross the porous membrane towards a chemoattractant. In another study, we identified in two melanoma cell lines new molecular partners of GSN, both in the cell nucleus and in a cytosolic fraction [7]. We also showed that GSN is present in relatively high amounts in several melanoma cell lines relative to cell lines representing other malignancies. Furukawa and colleagues detected truncated GSN form—GSNp85—lacking a C-terminal domain due to cleavage by caspase-8. The altered protein was co-expressed with wild-type GSN in normal tissue, but it was not observed in metastatic melanoma [8]. Fujita and colleagues demonstrated that GSN overexpression inhibited migration and acted as a metastasis suppressor in murine B16a melanoma cells. Importantly, it was shown that the C-terminal domain of GSN was responsible for these effects [9].

Melanoma is a very invasive tumor, for which early diagnosis and tumor resection before the occurrence of metastases give the best probability of a full recovery. Though there are several therapeutic approaches used in the treatment of melanoma, including targeting PD-L and BRAF kinase with V600E or V600D mutation, there is still no effective drug targeting all subtypes of skin melanoma nor ocular or mucosal melanoma or primary melanoma found in other organs [10,11]. Finding new better drugs or working combinations of existing drugs is crucial, especially that already a 1 mm thick primary tumor of skin melanoma is capable of giving metastases [12]. Therefore, because of that, further studies on melanoma biology are needed.

During melanomagenesis, transformed melanocytes undergo a transition from the radial growth phase (RPG) to the vertical growth phase (VPG) [13]. The former phase refers to a situation when the cells, upon losing control over their proliferation, grow in number and spread over the basement membrane (BM) of the skin. The latter describes the phase when melanoma cells acquire the ability to cross the BM and invade into the dermis, where they eventually reach blood vessels. Finally, melanoma cells intravasate the blood vessels to spread in the organism and, upon extravasation, form at distinct localizations metastatic sites. Thus, the moment of acquisition of the degrading ability of the BM is one of the critical points in melanomagenesis.

The skin’s BM, a manifestation of extracellular matrix (ECM), is composed mostly of collagen type IV, laminin, nidogen/enactin, perlecan, and heparin sulfate proteoglycan [14]. Melanoma cells, while traversing the BM, have to breakdown ECM. For this feature, invadopodia are responsible [15]. These protrusive structures are membranous invaginations rich in polymerized, i.e., filamentous (F-) actin, several signaling, scaffolding, and adhesion proteins, as well as matrix metalloproteases (MMPs), which are responsible for the digestion of ECM proteins [16]. While invadopodia are crucial for cells to protrude under 3-D conditions, there are other F-actin-rich structures taking part in both 2-D and 3-D movement. Lamellipodia, filopodia, and stress fibers are implicated here. All these structures possess diversely organized F-actin due to diversified sets of actin nucleators and their regulators [17,18]. The tightly regulated balance of these actin-based structures is responsible for the type of movement exhibited by cells: ameboid, mesenchymal, or mixed [19].

Our knowledge of the cell’s response to ECM remains insufficient. Therefore, because of that and because there is still a large gap in understanding the role of GSN in melanoma cells, we decided to evaluate the influence of GSN on the ability of A375 cells to move on the main fibrous constituents of the BM, i.e., laminin and collagen type IV. We extended our study to collagen type I and fibronectin, which are other ECM fibrous components of the skin and BM, respectively [20,21]. We additionally performed experiments on Matrigel-coated surface as this reagent resembles the composition of the BM [22,23]. For the purposes of this study, we decided to deprive the A375 cells of GSN production by means of the CRISPR/Cas9 (D10A) technique. We chose the A375 human melanoma cell line because it expresses high levels of GSN. We show here that the lack of GSN had the most severe effect on the A375 cells’ motility when the cells were grown on laminin. Moreover, we show that GSN-depletion tremendously reduces the number of cells able to invade the 3-D Matrigel. Altogether, we prove that GSN is an important player in A375 cells motility, and its role in melanoma biology should be further studied, especially in the context of protrusion on ECM proteins.

## 2. Materials and Methods

### 2.1. Cells and Culture Conditions

A375 cell line was obtained from the ATCC (ATCC**^®^** CRL-1619™). WM1341D and SK-MEL-28 cells were from Rockland Immunochemicals Inc. (Pottstown, PA, USA) and CLS GmbH (Eppelheim, Germany), respectively. Dulbecco’s modified Eagle’s medium with reduced concentration (1.5 g/L) of NaHCO_3_ (Polish Academy of Science, Wrocław, Poland), supplemented with 10% fetal bovine serum (FBS) (Thermo Fisher Scientific, Warsaw, Poland), 1% L-Glutamine (Thermo Fisher Scientific Warsaw, Poland) and 1% Antibiotic-Antimycotic (Thermo Fisher Scientific, Warsaw, Poland) was used to culture the cells. Cells were cultivated at 37 °C under a humidified atmosphere of 5% CO_2_ and subcultured twice a week.

### 2.2. Coating of the Plates and Dishes with ECM Proteins

Laminin-1 (Sigma-Aldrich, Poznań, Poland), fibronectin (Corning, New York, NY, USA), collagens type I and IV (Corning, New York, NY, USA), and Matrigel (Corning, New York, NY, USA) were used at a concentration of 1 μg/cm^2^. To coat the plates and coverslips with ECM proteins, the protein solution in Hanks’ Balanced Salt Solution (HBSS) (Thermo Fisher Scientific, Warsaw, Poland) was added to a cell culture dish. Then, 0.36 µg of protein per well in a 96-well plate, 2 µg of protein per well in a 24-well plate, and 9.6 µg of protein per well in a 6-well plate were added to obtain the concentration of 1 µg/cm^2^. That concentration is recommended by the manufacturer of laminin and fibronectin. The remaining proteins were used at the same concentration so that the results could be compared with each other. The plates and coverslips with the coating solutions were incubated at least for 4 h at 37 °C under a humidified atmosphere of 5% CO_2_. In the case of fibronectin and collagens types, I and IV, the coated surfaces were accordingly to the manufacturer dried after incubation for 45 min. Coated surfaces were washed three times with phosphate-buffered saline (PBS) before seeding the cells. Laminin, fibronectin, Matrigel, and collagen type IV are commercially available in their native form. Collagen type I requires prior preparation renaturation. The following solution was prepared to fulfill that: 4 parts of collagen type I, 5 parts of HBSS, 1 part of MEM 10× (Thermo Fisher Scientific, Warsaw, Poland), 1 part of 0.25 M NaHCO_3_, 2.65 parts of complete cell culture medium, and 0.3 parts of NaOH [24]. Prepared solutions of ECM proteins prior to the coating were kept on ice.

### 2.3. GSN Knockout with CRISPR/Cas9(D10A) Technique

A375 cells growing in a 35-mm plate were transfected after reaching 80% of the confluence with a mixture of two plasmids used in the CRISPR/Cas9(D10A) system. The double Nickase Plasmid system from Santa Cruz Biotechnology Inc. (Heidelberg, Germany) comprises coding sequences for nickase Cas9 (D10A), two different guideRNA (gRNA) sequences designed specifically for the gelsolin gene (sc-401005-NIC), and two cell selection methods, i.e., puromycin resistance and green fluorescent protein (GFP). The gRNAs’ sequences for GSN are as follows: 5′ccgggccgtgcagcaccgtg3′ and 5′atccagctgcacggtaaaga3′. For cell transfection, 2 mg/mL polyethyleneimine solution (Sigma-Aldrich, Poznań, Poland) was used in a ratio of 1:2.175 for 4 µg of DNA solution. Then, 24 h after transfection, the cells were seeded onto 15 cm plates. Cells were incubated with puromycin (Santa Cruz Biotechnology Inc., Heidelberg, Germany) at a concentration of 1 µg/mL. After forming the clones, they were then transferred individually using glass cylinders into a 24-well plate. The clones’ cultivation was continued with the usage of a selective antibiotic at a concentration of 0.5 µg/mL. After the stable lines were derived, they were verified for their correctness using immunocytochemistry, Western blot, and gDNA analyses to confirm the deprivation of GSN.

### 2.4. gDNA Analysis

gDNA was isolated using the DNA Purification Kit (EurX, Gdańsk, Poland) according to the manufacturer’s instructions and was used as a template in a PCR reaction performed with Color Taq PCR Master Mix (EurX, Gdańsk, Poland). PCR primers were designed to anneal to the sequences upstream and downstream from the sequences recognized by gRNA encoded by CRISPR/Cas9(D10A) plasmids (Fwd: 5′gtgcagccaggatgagag3′, Rev: 5′ccctgttactggtgcatc3′). PCR products were separated in a 2% agarose gel in Tris-acetate–EDTA (40 mM Tris pH 8.0, 20 mM acetic acid, 1 mM EDTA) buffer. The length of the amplified products was analyzed for *GSN*-knockout clones compared to control clones. Perfect 100 bp DNA ladder (EurX, Gdańsk, Poland) served as a marker.

To sequence the two alleles of the GSN-encoding gene, the altered CRISPR/Cas9(D10A) gene fragments were cloned into the pAcGFP-C1 plasmid using the NEBuilder HiFi DNA Assembly Master Mix. The gDNA of the *GSN* KO and CTRL KO clones served as a template for PCR reactions carried out using primers annealing upstream and downstream of the gRNA recognition sequences encoded by CRISPR/Cas9 (D10A) (Fwd: 5′gatctcgagctcaagcttcgaattctgaacagtgcagacctttg3′, Rev: 5′cgcggtaccgtcgactgcagaattcaattcaccagaacaggactaggc3′) and Phusion High-Fidelity DNA Polymerase (HF Buffer) (Thermo Fisher Scientific, Warsaw, Poland). Products of the PCR reactions were ligated with the pAcGFP-C1 plasmid, which was linearized with Eco*R*I. The *E. coli* DH5αstrain was transformed with the HiFi reactions. Plasmids selected on the basis of the analysis of restriction digestion were sequenced by Microsynth GmBH (Balgach, Switzerland).

### 2.5. Western Blot Analysis (WB)

Cells growing in the wells of a 6-well plate for 48 h were harvested and lysed on ice with the urea buffer (50 mM TRIS-HCl pH 7, 5% SDS, 8.6% sucrose, 74 mM urea, 1 mM DTT, 1:100 serine phosphatase inhibitor, 1:100 tyrosine phosphatase inhibitor) or cytoskeletal-bound protein extraction buffer (10 mM Tris-HCl pH 7.4, 100 mM NaCl, 1 mM ethylenediaminetetraacetic acid (EDTA), 1 mM ethylene glycol-bis(2-aminoethyl ether)-N,N,N**’**,N**’**-tetraacetic acid (EGTA), 1 mM NaF, 20 mM Na_4_P_2_O_7_, 2 mM Na_3_VO_4_, 1% Triton X-100, 10% glycerol, 0.1% SDS, 0.5% sodium deoxycholate) both with addition of 1:100 protease inhibitor cocktail. The protein concentration of the samples was measured using the Pierce BCA Protein Assay Kit (Thermo Fisher Scientific, Warsaw, Poland) or by the Bradford protein assay (SigmaAldrich, Poznań, Poland) according to the manufacturer’s instructions. Samples containing 30 µg of total protein were separated by SDS-PAGE in 12.5% polyacrylamide gel. The separated proteins were transferred to a nitrocellulose membrane by a semi-wet transfer method. Ponceau S nitrocellulose membrane staining was used to control protein loading and transfer efficiency. The membranes were blocked using 5% skimmed milk solved in TBS-T buffer (20 mM Tris, 150 mM NaCl:, Tween**^®^** 20 detergent: 0.1% (*w*/*v*)). To detect GSN, we used either mouse antibodies, clone GS-2C4 (Sigma-Aldrich, Poznań, Poland) at a dilution of 1:2000 or goat antibodies, clone C-20 (Santa Cruz Biotechnology Inc., Heidelberg, Germany) at a dilution of 1:200, while to detect GAPDH, we used mouse antibodies, sc-47724 (Santa Cruz Biotechnology Inc., Heidelberg, Germany) at a dilution 1:200. The secondary antibodies directed either against mouse (Cell Signaling) or goat (Santa Cruz Biotechnology, Heidelberg, Germany) were coupled to HRP and used at 1:4000 dilution. Both primary and secondary antibodies were diluted in the blocking solution. The immunoblots were developed using the Clarity Western ECL Substrate (Bio-Rad, Hercules, CA, USA), the chemiluminescence was detected with the ChemiDoc MP System (Bio-Rad, Hercules, CA, USA), and the results were analyzed using Image Lab 4.0 software (Bio-Rad, Hercules, CA, USA). PageRuler Prestained Protein Ladder, 10 to 180 kDa (Thermo Fisher Scientific, Warsaw, Poland) served as a marker.

All densitometry analyses were carried out on “1-sec exposure time” images using the “Line and Bands” tool of Image Lab software (Bio-Rad; Hercules, CA, USA). For densitometry of Ponceau S-stained membranes, intensity (volume) of whole protein loaded in the entire lane was measured. The volume of GSN bands, following normalization to Ponceau S staining, was normalized to loading control—GAPDH. Finally, obtained data for each clone were standardized to the mean intensity of the GSN signal from the cells grown on a non-coated surface.

### 2.6. Cell Proliferation Assay

To perform the BrdU 5-bromo-2-deoxyuridine (BrdU) cell proliferation assay, 2000 cells were seeded into ECM protein-coated wells of 96-well plates for 72 h before the experiment. The cell proliferation was measured using the BrdU Cell Proliferation Assay Kit (BioVision, San Francisco, USA) according to the manufacturer’s instructions. BrdU is a pyrimidine analog incorporated into the newly synthesized DNA of proliferating cells in the thymidine site. The method detects only proliferating cells by using anti-BrdU antibodies, secondary antibodies conjugated with HRP and the HRP substrate. Signal was measured at 450 nm using a microplate reader µQuant (BioTek Instruments Inc., Bad Friedrichshall, Germany).

### 2.7. Cell Confluence Analysis

Analysis of cells’ confluence over time was performed using IncuCyte Live-Cell Analysis Imaging System. The images from the spontaneous migration assay were used. Label-free phase segmentation analysis on each from 2 h interval photos was performed. The confluence mask was set to both exclude background and include all cells visible in the field of view. The growth of cells over time was measured automatically by IncuCyte System based on the set confluence mask. The data was reported as the percentage of reached confluence over time for 72 h.

### 2.8. Immunocytochemistry and Fluorescence Microscopy

Immunocytochemical analyses were performed as described elsewhere [25]. The 4% formaldehyde in PBS was used to fix the cells growing on coverslips. Next, the cells were permeabilized with 0.1% Triton X-100 in PBS at RT for 6 min and blocked with 1% BSA/PBS solution at RT for 30 min. The coverslips were incubated overnight at 4 °C with primary antibodies diluted in blocking solution. We used mouse anti-GSN antibodies (clone GS-2C4) (Sigma-Aldrich, Poznań, Poland) at 1:200 dilution followed by staining with donkey anti-mouse-Alexa Fluor 488 or 568 (Thermo Fisher Scientific, Warsaw, Poland) at 1:200 dilution. In order to detect F-actin and cell nucleus, we used phalloidin-Alexa Fluor 568 (Thermo Fisher Scientific) at 1:200 dilution and Hoechst 33342 (Thermo Fisher Scientific, Warsaw, Poland) at 1:1000 dilution. The coverslips were mounted with Dako Fluorescent medium (Clontech, Heidelberg, Germany). Photos were taken using Leica TCS SP8 Confocal Laser Scanning Microscope or Leica Stellaris Microscope with the Lightning module and then analyzed with Leica Application Suite X (LAS X) (Leica, Wetzlar, Germany).

The distribution ratio of cells with different GSN levels was done based on the photos with the visualization of immunostaining against GSN and calculated using the “Over-/Underexposure” tool of the LasX software (Leica, Wetzlar, Germany). The tool assigns a green color to underexposed areas in the image and blue color to overexposed areas in the image. The cells that were generating a blue signal indicating that the sample was overexposed were classified as cells with a high level of GSN expression. The cells showing an intense red color were characterized as expressing GSN at a medium level. While the cells with a predominance of black color were defined as producing GSN at a low level. For each cell line, 10 pictures were taken without magnification using a 60× oil immersion lens of Leica TCS SP8 Confocal Laser Scanning Microscope. Images were acquired with the same settings. At least 159 cells were analyzed for each cell line.

### 2.9. Structured Illuminated Microscopy (SIM)

SIM was performed with an Elyra 7 with Lattice SIM microscope with a 63× oil immersion Plan-Apochromat NA1.4 objective (Zeiss, Jena, Germany). Samples were illuminated with the following laser lines: 488 and 568, and dichroic LBF 405/488/561/642 was used. Fluorescence was collected by an sCMOS pco-edge 4.2M camera. After acquisition, raw images containing 13 phase-shifted were reconstructed with ZEN black software (Zeiss, Jena, Germany). For the SIM analysis, the cells were stained with rabbit anti-cortactin antibodies (sc-11408) Santa Cruz Biotechnology Inc., Heidelberg, Germany) at 1:200 dilution, followed by staining with donkey anti-rabbit-Alexa Fluor 488 (Thermo Fisher Scientific, Warsaw, Poland) at 1:200 dilution and phalloidin-Alexa Fluor 568 to detect F-actin.

### 2.10. Projected Cell’s Area Assessment

The projected cells’ area assessment was done based on the cell photos acquired with SIM. The projected area of cells was measured automatically using ImageJ software. Threshold was adjusted to the border of the cell and then the area was measured using “analyze particles” analysis. The data were presented as a mean cell area. Thirty cells per group of clones were analyzed.

### 2.11. Stress Fiber Number Evaluation

The assay is described elsewhere [25]. Briefly, the cells seeded onto coverslips coated with different ECM proteins were stained with Alexa Fluor 488 phalloidin to visualize filamentous actin as a basic component of stress fibers structure. Although stress fibers are usually defined as 10–30 actin filaments mass, here we decided to calculate only thick bundles of stress fibers, identified as phalloidin-positive linear structures with the highest fluorescent intensity. The stress fibers’ bundles composed of a higher number of actin filaments are thick and more stable, resulting in worse cell movement properties. For this reason, the histogram of fluorescence was plotted over the cell length firstly. Then the number of bundled stress fibers per cell was identified as the number of peaks representing the intensity value higher or equal to 75% of the highest fluorescence peak. The photos were taken using Zeiss 7 with Lattice SIM with Zen black software (Zeiss, Jena, Germany). Analysis of stress fibers’ number was done using Zeiss ZEN software (blue edition, version 3.3.). The data were presented as the mean number of stress fibers formed per cell.

### 2.12. Filopodia Number, Length, and Density Evaluation

For filopodia analysis, cells were seeded onto coverslips coated with different substrata; 48 h later, the cells were fixed and stained with phalloidin-Alexa Fluor 488. The photos of phalloidin-stained cells were taken using Zeiss Elyra 7 with Lattice SIM with Zen Black software (Zeiss, Jena, Germany). The data analysis was performed with the ImageJ plugin FiloQuant (Single Image FiloQuant) (ImageJ, F. Cordelieres, Institute Curie, Paris, France), allowing for detection and measurement of filopodia-like protrusions of cells [26]. Thirty cells per group were analyzed. The data were presented as the mean number, length, and density of filopodia per cell. The density of filopodia was calculated as the ratio of the filopodia number and the length of the cell edge as described elsewhere [27].

### 2.13. Invadopodia Number and Size Calculation

Analysis of the number and the projected area of invadopodia of CTRL KO and *GSN* KO cells cultured on different substrata was performed based on the photos with the immunostaining against cortactin. Photos of cells were taken using Zeiss Elyra 7 with Lattice SIM with Zen Black software (Zeiss, Jena, Germany). The size of invadopodia was measured manually with the “Closed Bezier” from graphic tools in Zeiss ZEN (blue edition, version 3.3.). Thirty cells per group were analyzed. Data were presented as a mean number of invadopodia per cell and their mean projected area.

### 2.14. Invasion Assay

The assay is described elsewhere [28]. Briefly, the cells 24 h after serum starvation were seeded in serum free-medium onto the Transwell filters (BD Biosciences, Wroclaw, Poland) coated with Matrigel (BD Biosciences, Wroclaw, Poland). The 20% FBS was used as a chemoattractant. After 24 h, the cells that traversed the Matrigel layer to the lower side of the insert membrane were fixed and stained with Hoechst 33342, and on the base of detected cells, nuclei counted under a fluorescent microscope.

### 2.15. Cell Migration Assays

Spontaneous migration assay and collective migration assay were performed using IncuCyte Live-Cell Analysis Imaging System (Sartorius, Praha, Czech Republic). Wells of 96-well plates (IncuCyte ImageLock, Sartorius, Praha, Czech Republic) were coated with ECM proteins. For spontaneous migration assay, 1000 cells per well were seeded into each well, and then they were incubated in IncuCyte Live Cell Analysis Imaging System with taking photos every 2 h for 72 h. Collected photos were analyzed with a Manual Tracking plugin (ImageJ, F. Cordelieres, Institute Curie, Paris, France). Three parameters describing cell migrative properties (the cell trajectory, covered distance, velocity, and directionality) were determined as described elsewhere [25]. Ninety cells per group were analyzed (30 cells per clone). For collective migration assay, 40,000 cells per well were seeded into 96-well IncuCyte ImageLock plates. For Matrigel and laminin-coated surface, 100,000 and 120,000 cells were seeded, respectively, as the cells covered a smaller area under these conditions. After reaching cell confluence, wounds in the cell monolayers were made using the Wound Maker. The plate was transferred into IncuCyte Live Cell Analysis Imaging System and incubated for 72 h with taking photos every 2 h. The collective migration analysis was performed with IncuCyte software, and the data was presented as a percent of scratch overgrown by cells over time. Six photos per clone were analyzed.

### 2.16. Gelatin Digestion Assay

The procedure was performed as described elsewhere [29]. Briefly, coverslips coated with Poly-L-Lysine (Corning, New York, NY, USA) in a 24-well plate were washed with PBS, fixed with 0.5% glutaraldehyde for 15 min at RT, and washed again with PBS. Immediately after that, the coverslips were incubated on a drop of 0.2% gelatin-fluorescein solution ( Thermo Fisher Scientific, Warsaw, Poland) in the dark for 10 min at RT, followed by incubation in 5 mg/mL sodium borohydride solution at RT and washing with PBS. After additional washing of the coverslips with cold cell culture medium, 30,000 cells were seeded onto each gelatin-fluorescein-coated coverslip and then they were incubated in a full cell culture medium at 37 °C, 5% CO_2_ for 12 h. After that, cells were fixed with 4% formaldehyde for 20 min at RT and stained using phalloidin-Alexa Fluor 568 and Hoechst 33342 (Thermo Fisher Scientific, Warsaw, Poland) to detect F-actin and cell nuclei, respectively. Photos were taken using Leica TCS SP8 Confocal Laser Scanning Microscope with Leica Application Suite X (LAS X, Leica, Wetzlar, Germany) software. Three parameters were analyzed using the Fiji software: the number of formed invadopodia per cell, the projected area of cells, and the percentage of gelatin-digested area per projected area of a single cell. Thirty cells per group of clones were analyzed, and data were reported as mean + SD.

### 2.17. Statistical Analysis

The analyses were performed on the data obtained from three CTRL KO and three *GSN* KO clones in each experiment, and they acted as biological replicates. Both graphs and statistical analyses were performed using GraphPad Prism 7 and 8 (GraphPad Software Inc., San Diego, CA, USA). Data shown on graphs were presented as a mean ± SD. The outlier points, if occurred, were detected and eliminated using the 2 standard deviations (2SD) method. Outliers were identified as any values lying two standard deviations below and above the mean. The first step of statistical analysis was the verification of the normality of data distribution using Shapiro–Wilk’s normality test. Further analyses of statistical data significances were performed with nonparametric and parametric versions of unpaired Student’s t-test or one-way and two-way ANOVA with post hoc test (Dunn’s or Sidak’s multiple comparisons test). The significance levels were set as *p* < 0.05 (*), *p* < 0.01 (**), *p* < 0.001 (***) and *p* < 0.0001 (****).

## 3. Results

### 3.1. GSN Is Found in Motile Structures of A375 Cells When Cultured on ECM Proteins, and GSN Expression Is Not Homogenous in Melanoma Cell Lines

**Figure 1:**
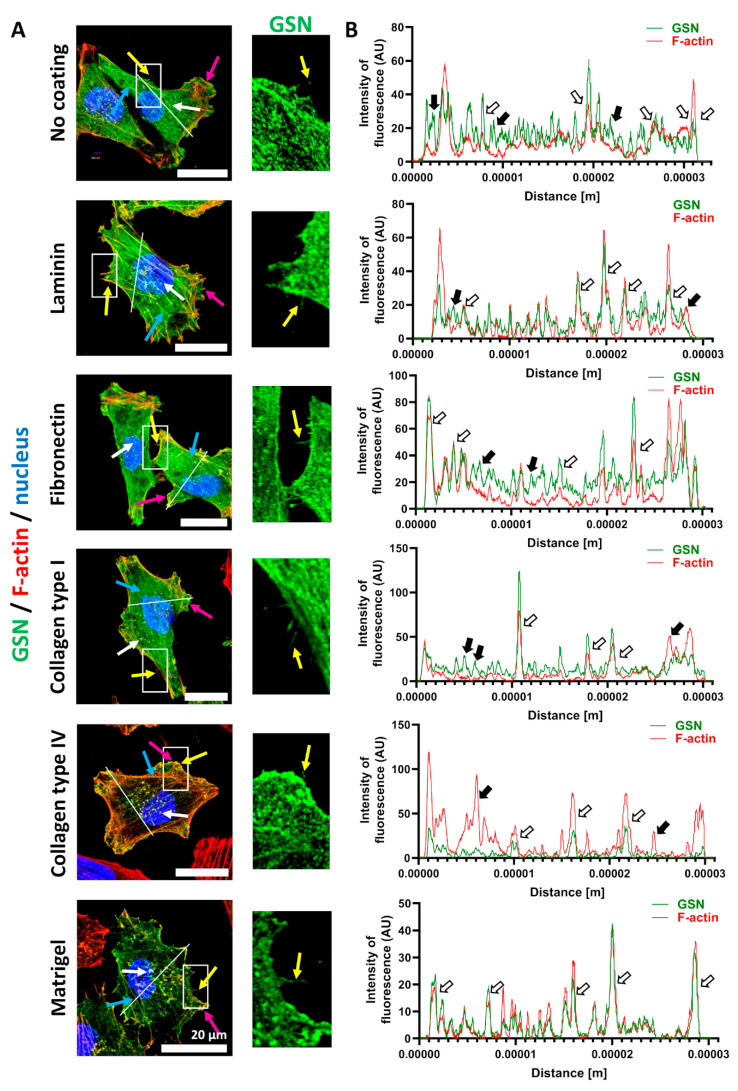
GSN is present in motile structures of A375 cells growing on different ECM proteins. (**A**) The cells were seeded onto ECM proteins. Forty-eight hours later, the cells were fixed and stained to detect GSN, F-actin, and nuclei. White arrows indicate invadopodia, pink arrows lamellipodia, yellow arrows filopodia, and blue arrows stress fibers. The insets marked with white rectangle silhouettes show the enlarged areas of the representative cells with detected GSN, demonstrating that this protein is present in the filopodia. Scale bar: 20 μm. A line was drawn on a merged photo of the representative cell, and the fluorescence histograms representing signal intensities for Alexa Fluor 488 and 568 fluorochromes were plotted on this basis (**B**). White and black arrows indicate the overlapping and non-overlapping fluorescence intensity peaks for GSN and F-actin, respectively.

First, we tested whether gelsolin’s subcellular distribution is changed in A375 cells when grown on selected ECM fibrous proteins. In our experiments, we studied laminin, fibronectin, collagens type I and IV, and Matrigel, which represents the composition of the skin’s BM, which has laminin (~60%), collagen IV (~30%), entactin (~8%), and the heparin sulfate proteoglycan perlecan (~2–3%) as its four major components [30]. Upon 48 h of cells cultivation on ECM proteins, the cells were fixed and stained to detect GSN and F-actin, as GSN is primarily an actin-binding protein. We found that GSN co-localized with F-actin in multiple structures, including filopodia, lamellipodia, and invadopodia, structures rich in F-actin and crucial for cells’ migratory abilities (Figure 1A). Moreover, GSN aligned along stress fibers. Analysis of the histograms for fluorescence intensity showed that in the cells growing on every tested ECM protein, the peaks for GSN and F-actin fluorescence intensities were usually overlapping (Figure 1B). Interestingly, for the cells seeded onto laminin and Matrigel, that overlap was more complete.

Taken together, we can state that GSN can be found in F-actin-rich structures involved in the motility of A375 cells.

**Figure 2:**
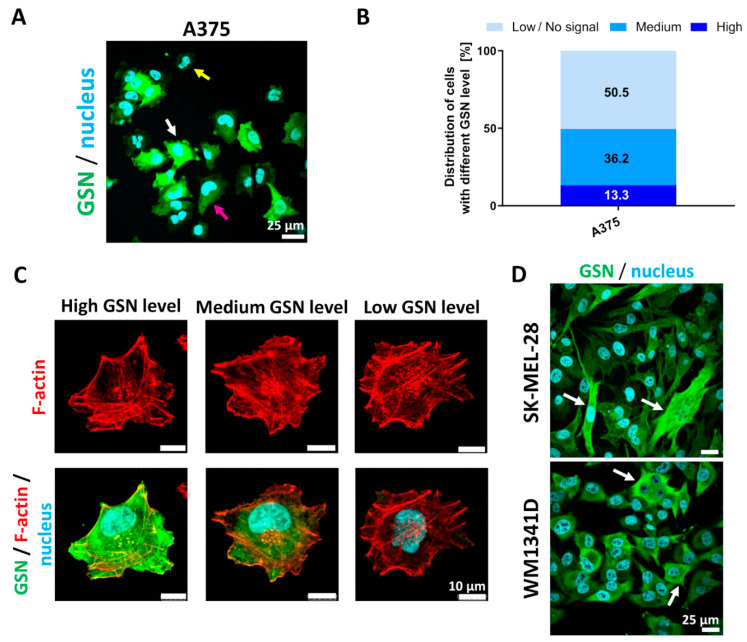
Phenotypic heterogeneity in terms of GSN level in melanoma cell lines. (**A**) The A375 cells growing on a non-coated surface were fixed and stained to detect GSN and cell nuclei. The white arrow indicates a cell with a high GSN level, the pink arrow a cell with a medium GSN level, and the yellow arrow a cell with a low GSN level. (**B**) Estimation of the distribution of cells with high, medium or low GSN levels within the A375 cells population. The percentage for every group is presented on a given section of the bar (10 images and at least 159 cells were analyzed). (**C**) F-actin subcellular localization in cells with different GSN levels. The cells seeded onto a non-coated surface were fixed and stained with fluorescently labeled phalloidin, anti-GSN antibodies, and Hoechst 33342. Scale bar: 10 μm. (**D**) Immunostaining of other melanoma cell lines, i.e., SK-MEL-28 and WM1341D with antibodies directed against GSN and Hoechst 33342 to detect GSN and cell nuclei. The white arrows indicate cells with high GSN levels. Scale bar: 25 μm.

Interestingly, we noted that A375 cells are heterogenous in terms of GSN level. Immunocytochemical staining of A375 cells revealed that cells could exhibit high, medium, and low levels of GSN (Figure 2A). Based on the fluorescence images, we calculated the distribution of cells with varying levels of GSN (Supplementary Figure S1). Half of the cells have a low or very low level of GSN, while 36% of the cells exhibit a medium level of GSN, and just 13.3% of the cells have a high GSN level (Figure 2B). As GSN is an actin severing protein, we investigated next the F-actin organization of the cells with high, medium, and low GSN levels. Surprisingly, we found that the morphology of these cells and the F-actin distribution were similar despite a reduced GSN level (Figure 2C). Finally, we looked at whether this phenomenon is characteristic only for A375 cells. We immunostained the SK-MEL-28 and WM1341D cells to detect GSN. Similar to A375 cells, these two melanoma cell lines were isolated from primary tumor sites. Both cell lines comprise cells with differing GSN levels (Figure 2D).

### 3.2. Knockout of GSN in Melanoma A375 Cells

**Figure 3:**
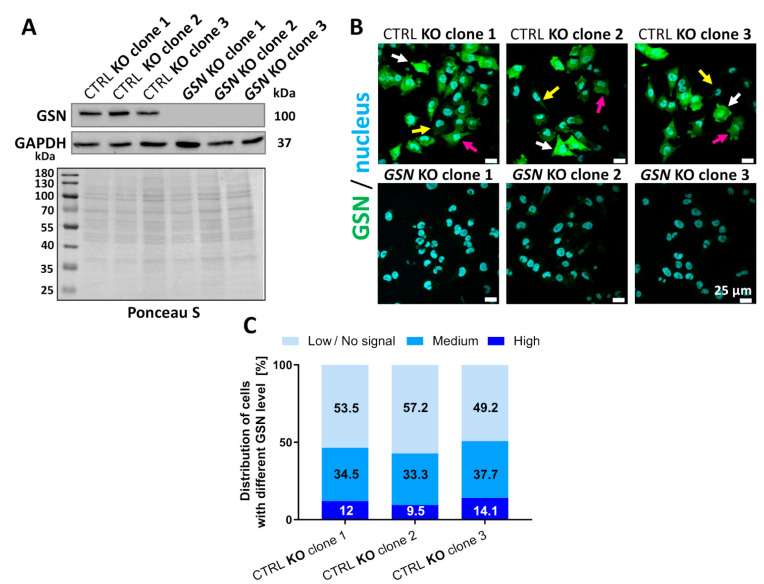
Successful knockout of the *GSN* gene using the CRISPR/Cas9 (D10A) system. (**A**) Western blot analysis of the A375 clones’ lysates. The membrane was incubated with mouse antibodies detecting GSN and GAPDH as a loading control. Additionally, the membrane was stained with Ponceau S. Thirty micrograms of protein were loaded into every well. (**B**) Immunocytochemical analysis of the clones using mouse antibodies directed against GSN. Note that the control clones are heterogenous in terms of GSN level. White arrows indicate cells with a high GSN level, pink arrows cells with a medium GSN level, and yellow arrows cells with a low GSN level. Images were acquired with the same settings. Scale bar: 25 μm. (**C**) Estimation of the distribution of the cells with high, medium, or low GSN levels within the cell populations of three control clones. The percentage for every group is presented on a given section of the bar (10 images and at least 159 cells were analyzed).

To study the role of GSN in the motility of A375 cells growing on different ECM proteins, we decided to knockout the *GSN* using the CRISPR/Cas9(D10A) technique. Previously, with the double nickase system (control double nickase plasmids) coding for non-targeting 20 nt scramble guide RNA sequences, we have obtained three control A375 clones, which we named CTRL KO clones [25,31]. For the purpose of this research, we generated stable A375 clones with *GSN* knockout using the same double nickase system. We obtained several clones resistant to puromycin and with changed gDNA in the *GSN* region (Supplementary Figure S2A). Some of these were GSN-negative in the Western blot analysis (Supplementary Figure S2B). Finally, for further analyses, we selected three clones, which we named *GSN* KO (Supplementary Figure S2C), in which Western blot and immunocytochemical analyses showed no signal for GSN (Figure 3A,B). While performing further experiments, we noted that the second clone exhibited some low amounts of gelsolin (Supplementary Figure S3A,B). However, in comparison to control clones, its level was so low that we could not perform any comparative densitometric analysis because it was impossible to set an exposure time giving sufficient signal for the GSN band for *GSN* KO clone no. 2 and non-overexposed signals for control clones. It is crucial to note here that for the WB analyses, we took two different antibodies directed against GSN; one of them recognizes the second half of GSN [8], while the goat polyclonal antibody recognizes the last 20 residues of the C-terminus [8]. We chose these two antibodies because gene editing takes place at the *GSN* region corresponding to the N-terminus of GSN. Should there be any product at the protein level in the cells with CRISPR/Cas9-edited *GSN*, both antibodies used here would detect it. Additionally, upon completion of the set of experiments presented here, we again subjected the *GSN* KO clone 15 to the immunocytochemical analysis. As presented in Supplementary Figure S3C, the level of GSN in that clone was undetectable when the images were acquired at the same settings.

We additionally analyzed the gDNA adjacent to the edited region of GSN in all clones by sequencing the PCR products cloned into a pAcGFP-C1 plasmid. Here we consider two different PCR products cloned into the plasmid as two alleles. This analysis revealed that in the case of one allele of *GSN* KO clone no. 1 and one allele of *GSN* KO clone no. 2, though the nucleotide sequences were edited, open reading frame (ORF) was not out-of-frame (Supplementary Figure S4). Although we detected very low amounts of GSN for *GSN* KO clone no. 2 (Supplementary Figures S3A,B and S5), we did not detect any signal for GSN in the case of the *GSN* KO clone no. 1 (Supplementary Figure S5). It is crucial to note that all *GSN* KO clones gave comparable outcomes in experiments presented in this study. Thus, we conclude that the scant level of GSN in the *GSN* KO clone no. 2 is neglectable. In the following analyses, we present pooled results for three clones per condition. However, in order to show the distribution of the results obtained for different clones within the pooled results, we present the outcomes for every clone in different colors (wherever possible).

We again observed phenotypic heterogeneity concerning GSN level in control clones. Immunocytochemical analysis of three control clones revealed the presence of cells with high, medium, or low GSN levels (Figure 3B). We calculated the distribution ratio of the cells with varying GSN levels. Similar to the results presented for A375 cells, the percentage of the cells with low/no signal for GSN was between 49% and 57%, while there were 33–37% of the cells with medium GSN levels and only 9–14% of the cells with GSN levels (Figure 3C).

### 3.3. GSN Knockout Does Not Change the Proliferation Rate of A375 Cells

**Figure 4:**
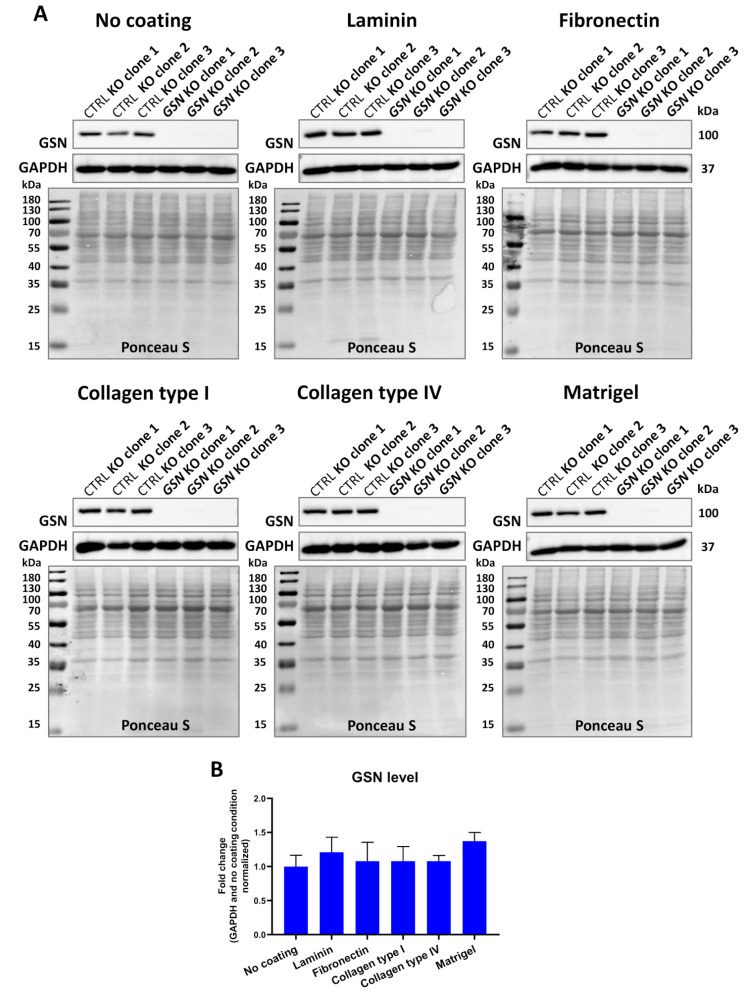
*GSN* knockout does not influence the expression level of GSN in A375 cells. (**A**) Western blot analysis of the A375 cells lysates obtained from the cells growing on ECM protein for 48 h. The membranes were stained with Ponceau S to analyze the total content of proteins on the lanes. Next, they were probed with anti-GSN antibodies and finally reported to detect GAPDH, which served as a loading control. Thirty micrograms of protein were loaded into every well. (**B**) Densitometric analysis of the results presented in (**A**) for control cells (*n* = 3). Results are expressed as the mean ± SD; ordinary one-way ANOVA with post hoc (Dunnett’s multiple comparisons) test.

For the purpose of this research, we decided to analyze several A375 cell parameters connected with motility. Outcomes obtained for the cells growing on ECM protein coatings were compared to the results for the cells growing on a non-coated surface. First, we checked whether culturing the cells on different ECM proteins changes the expression level of GSN. To assess the level of GSN, a Western blot analysis was performed. We noted that culturing the cells for 48 h on the ECM proteins studied here did not change the level of GSN in control cells (Figure 4A,B).

**Figure 5:**
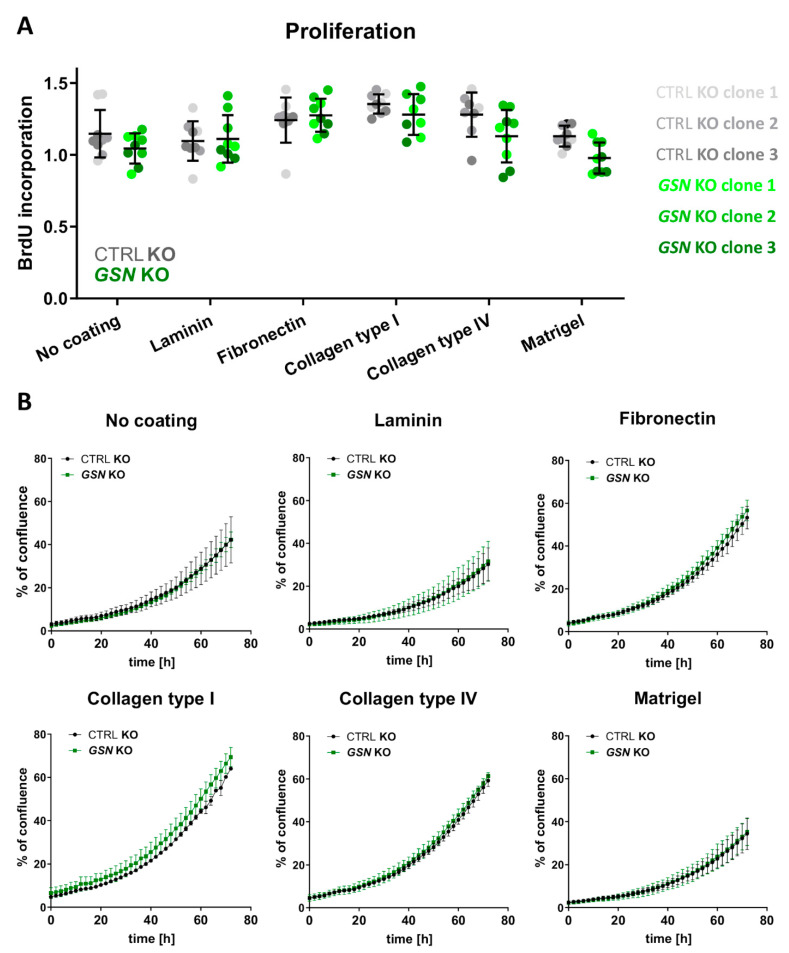
*GSN* knockout does not influence the proliferation of A375 cells. (**A**) BrdU assay performed on cells growing on different ECM proteins for 72 h (*n* = 8–9); unpaired t-test. (**B**) The cells’ confluence was assayed with the help of the IncuCyte system. The cells seeded into ECM proteins-coated wells of 96-well plate were photographed every 2 h over 72 h. Next, the confluence was calculated (*n* = 3); two-way ANOVA with Sidak’s multiple comparisons test. Results are expressed as the mean ± SD.

We also looked at the proliferation rate of A375 cells with *GSN* knockout for two reasons. First, we wanted to test whether GSN is crucial for cell proliferation, and second, we wanted to know if changes in proliferation rate could affect the outcomes from, e.g., the analysis of collective migration. We used the BrdU assay, which measures the ability of the cells to incorporate a thymidine analog into newly synthesized DNA. That analog is later labeled and finally detected. We found out that GSN depletion did not change the proliferation rate of A375 cells despiteless of the type of ECM protein (Figure 5A). We also analyzed control and GSN-depleted clones separately. We observed that control cells growing on collagen type I were better proliferating in comparison to the non-coated surface (Supplementary Figure S6). In the case of *GSN* KO cells, we noted an increased number of cells not only when growing on collagen type I but also fibronectin.

Next, we assessed the confluence of the cells growing on selected ECM proteins over 72 h with the help of the IncuCyte system. There were no statistically significant differences in overgrowing the surfaces coated with ECM proteins between control and *GSN* KO clones (Figure 5B). Interestingly, we noted differences in the overgrown areas when the control and *GSN* KO cells were analyzed separately. Control cells covered the surface faster when they grew on collagens type I and IV and fibronectin (Supplementary Figure S7A). However, control cells overgrew the surface slower when grown on laminin. The same phenomenon was observed for the cells not producing GSN, but here we also noted slower filling of the space during the last 12 h for cells attached to Matrigel (Supplementary Figure S7). Exemplary images acquired 72 h after seeding the cells are shown in Supplementary Figure S7B.

Altogether, we found that depletion of GSN production in A375 cells did not alter their proliferation abilities. Additionally, we observed that seeding A375 cells on collagen type I improved the proliferation abilities of these cells, which was mirrored in the highest surface overgrowing rate. Moreover, both types of clones covered the surface to a greater extent when attached to collagen type IV and fibronectin. On the contrary, A375 cells covered the surface area at the smallest extent when seeded on laminin.

### 3.4. Control and GSN KO A375 Cells Have Changed Morphology and Formation of F-Actin-Rich Protrusive Structures When Growing on Some ECM Proteins

**Figure 6:**
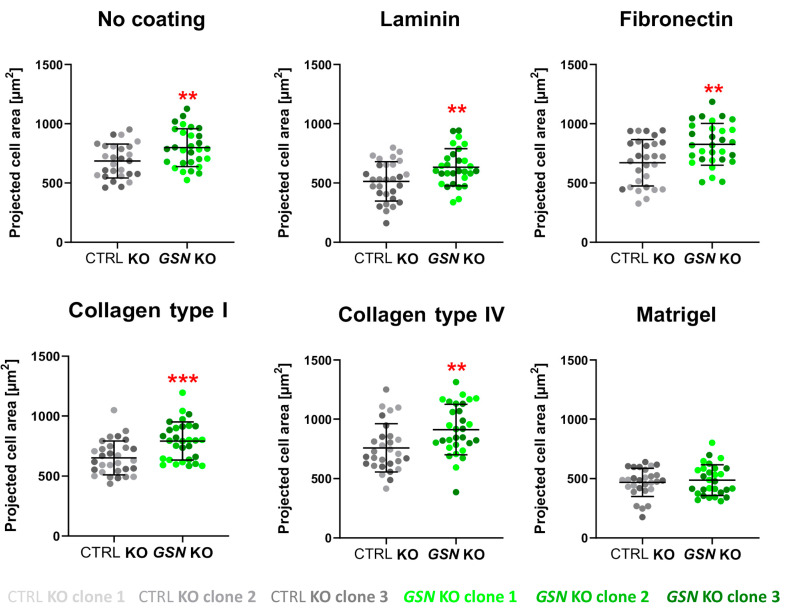
GSN-deprivation changes the projected cell area. On the basis of the photos taken 48 h after seeding the cells onto coated coverslips with selected ECM proteins (presented in Figure 7), the projected cell’s area was evaluated using ImageJ as described in the materials and methods section (*n* = 30). Results are expressed as the mean ± SD; *p* ≤ 0.01 (**), and *p* ≤ 0.001 (***); unpaired t-test and Mann-Whitney test.

Next, we evaluated the projected cell’s area for both types of clones growing on different ECM proteins. We detected differences for this parameter when we compared clones without GSN to control cells (Figure 6). Only for the cells growing on Matrigel were there no differences in the surface area. Otherwise, the *GSN* KO cells were occupying more of the coverslips’ surface in comparison to control cells. When we analyzed control and *GSN* KO cells separately, we noted that the cells growing on laminin and Matrigel in both cases occupied less space on the surface (Supplementary Figure S8). Additionally, we noted only for control cells growing on collagen type IV that they were more spread on the substrate than the cells growing on the non-coated surface.

**Figure 7:**
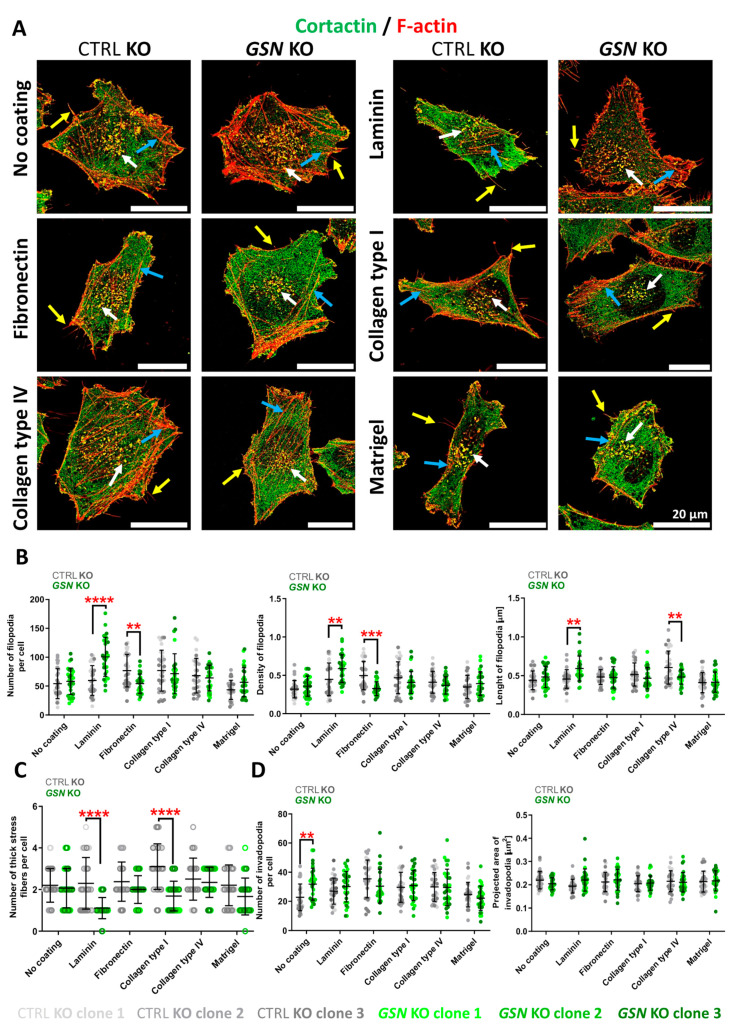
GSN is important for filopodia and thick actin bundles formation in A375 cells growing on laminin. (**A**) The cells incubated for 48 h on studied ECM fibrous proteins were fixed and stained appropriately to detect cortactin and F-actin. Images of the cells were taken using SIM microscopy. Yellow arrows highlight filopodia, white arrows invadopodia, and blue arrows thick actin bundles. Scale bar: 20 μm. (**B**) Based on the captured images, the number, density, and length of filopodia were assessed (*n* = 30). (**C**) The number of thick actin bundles was evaluated on the basis of cells microphotographs shown in A (*n* = 30). (**D**) Invadopodia were calculated on the basis of photos shown in A (*n* = 30). Results are expressed as the mean ± SD; *p* ≤ 0.01 (**), *p* ≤ 0.001 (***) and *p* ≤ 0.0001 (****); two-way ANOVA with Sidak’s multiple comparisons test.

Next, we evaluated the morphology of the cells growing on the studied ECM proteins. We analyzed the additional formation of filopodia, stress fibers, and invadopodia, as these structures are involved in cell motility [32]. To be able to assess the number, projected area, or density of these subtle structures, we employed structured illuminated microscopy (SIM) to increase the resolution of acquired images. Microphotographs were of sufficient resolution to quantitatively evaluate filopodia, thick actin bundles, and invadopodia (Figure 7A). The cells were stained for F-actin and cortactin, a marker of invadopodia [33].

We showed that filopodia formation in GSN-deficient cells was much improved when grown on laminin when compared to control cells. Additionally, filopodia formed under these conditions were also denser and longer (Figure 7A,B). The opposite effect was observed in cells growing on fibronectin, where cells lacking GSN expression produced fewer filopodia which were less dense compared to the control cells. When we compared the cell groups separately, the control clones formed more filopodia on the surface coated with fibronectin and collagen type I than on the non-coated surface. In contrast, cells growing on laminin lacking GSN produced more filopodia than when growing on a non-coated surface (Supplementary Figure S9A).

Next, we noted that A375 cells growing on collagen type IV exhibited a higher number of thick actin bundles (Supplementary Figure S9B). Interestingly, when we compared the cells without GSN to control cells, we observed that the number of these structures was significantly decreased in the case of collagen type I-coating (Figure 7A,C). Moreover, the same effect was observed for laminin-coating. Finally, we evaluated the formation of invadopodia. Indeed, cortactin was present in F-actin-rich spots localized in the vicinity of the cell nucleus (Figure 7A), confirming their invadopodial nature. A375 cells formed a higher number of invadopodia when cultured on fibronectin and collagens (Supplementary Figure S9C), but there were no changes in regard to the projected invadopodia area. Intriguingly, in the case of *GSN* KO cells, we noted diminished invadopodia number on Matrigel when compared to the no coating condition (Supplementary Figure S9C). We noted changes between control and *GSN* KO clones only when the cells were incubated on the non-coated substrate (Figure 7D). We did not notice any changes for the projected invadopodia area.

In summary, it is clear that GSN-depletion has a major effect on filopodia and thick actin bundles formation when the cells are grown on laminin. However, invadopodia under this condition remain unaffected by GSN-deprivation.

### 3.5. GSN-Depletion Results in Impaired 2-D Migration of A375 Cells on Selected ECM Proteins

**Figure 8:**
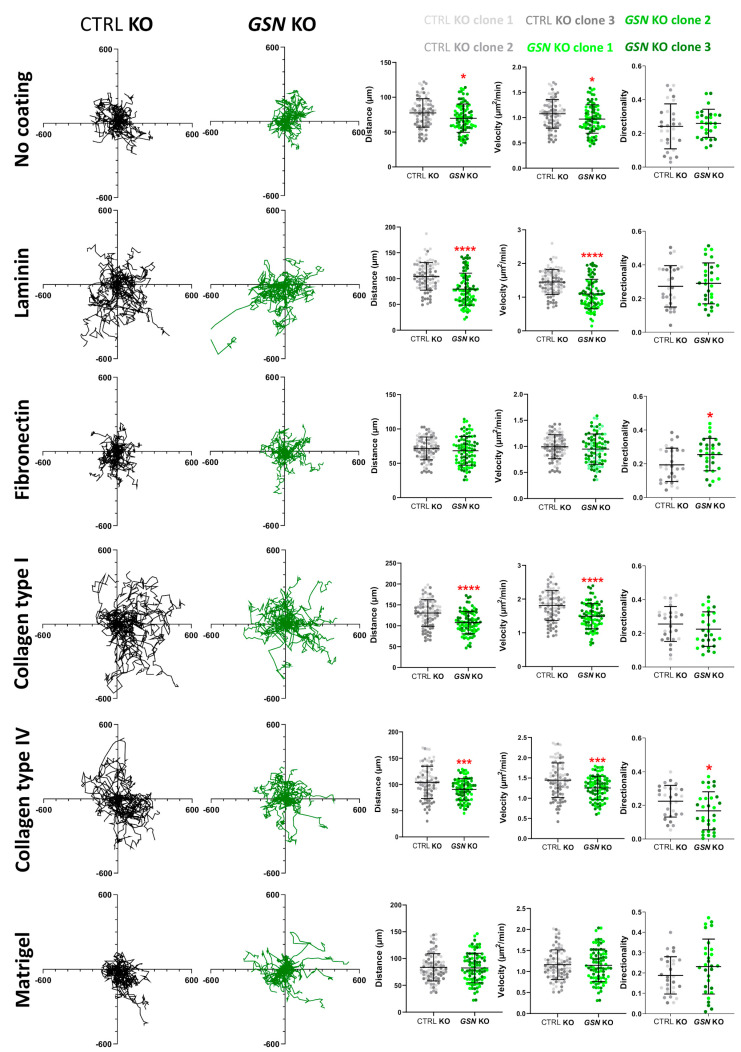
Spontaneous migration is heavily impaired in A375 cells not producing GSN in the case of laminin and collagens type I and IV coating. The cells were seeded into coated wells with different ECM proteins of a 96-well ImageLock plate. For 72 h hours, the cells were monitored with the IncuCyte system. Next, trajectories of single cells (*n* = 90) were plotted, and distance (*n* = 90), velocity (*n* = 90), and directionality (*n* = 30) of moving cells were assessed. Results are expressed as the mean ± SD; *p* ≤ 0.05 (*), *p* ≤ 0.001 (***) and *p* ≤ 0.0001 (****); unpaired t-test and Mann-Whitney test.

As we noticed changes in the formation of F-actin-rich structures between control cells and *GSN* KO clones, next, we evaluated the ability of the studied cells to protrude on a 2-D surface. First, we focused on the spontaneous ability of the cells to protrude. We monitored the cells for 72 h with the IncuCyte system and based on the tracks recorded for single cells (Figure 8), we calculated the distance, velocity, and directionality of cell movement. We noted that the distance covered and thus the velocity of A375 cells were increased for control cells when they grew on laminin and collagens (Supplementary Figure S10A,B). Next, we observed that on the non-coated surface, distance and velocity were diminished for *GSN* KO cells when compared to control clones (Figure 8). However, the most significant decrease in distance covered by the *GSN* KO cells (and thus velocity) was noted for laminin- and collagen-coatings. However, diminished directionality was observed solely for the collagen type IV-coating. In the case of seeding the cells onto fibronectin, we observed surprisingly improved directionality of the cells devoid of GSN. Strikingly, we did not note any differences between the control cells and *GSN* KO when grown on Matrigel.

**Figure 9:**
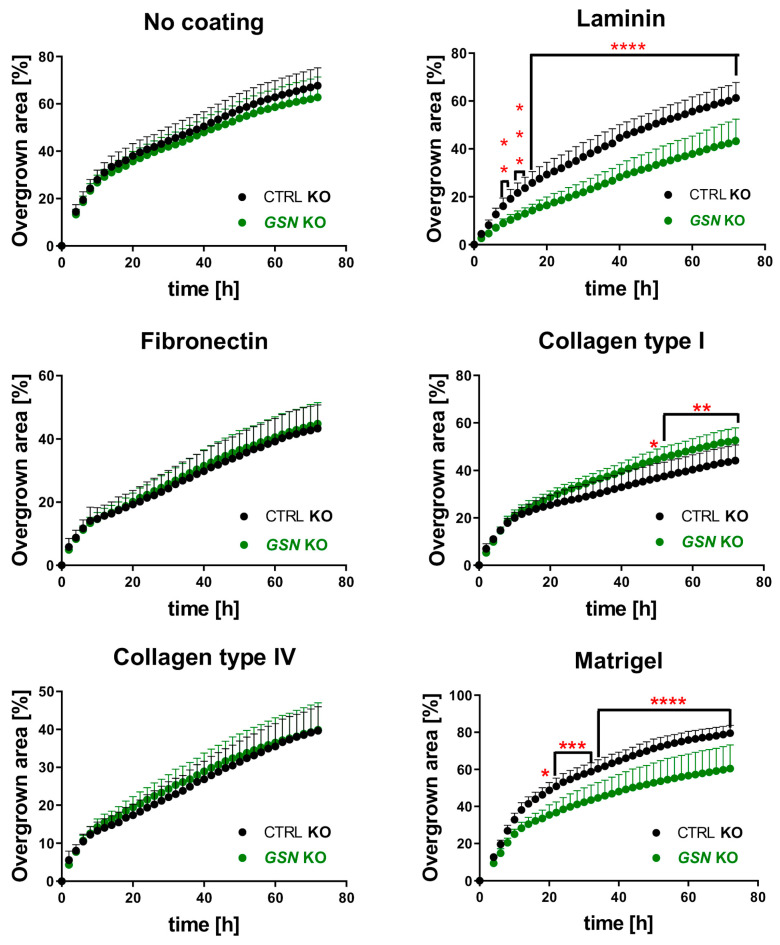
GSN is important for the collective migration of A375 cells on laminin and Matrigel. Upon making the gap with the Wound Maker, the cells were monitored for 72 h. A photo was taken every 2 h. Next, the percentage of overgrown scratches by the cells over time was calculated using the IncuCyte software (*n* = 9). Results are expressed as the mean ± SD; *p* ≤ 0.05 (*), *p* ≤ 0.01 (**), *p* ≤ 0.001 (***) and *p* ≤ 0.0001 (****); two-way ANOVA with Sidak’s multiple comparisons test.

Next, we looked at the ability of the cells to migrate collectively. With the help of the wound maker, we obtained standardized scratches, and for 72 h we monitored the cells with the IncuCyte system to test how fast they close the gaps. Here, we also assessed the ability of the cells to close the gap separately for control and GSN-depleted cells. We noted that control cells closed the scratch more quickly on Matrigel when compared to the “no coating” condition (Supplementary Figure S11A). Whereas in the case of cells cultured on laminin, fibronectin, and collagens, the A375 cells closed the gap slower in comparison to the situation of cells growing on the non-coated surface (Supplementary Figure S11A). We noted statistically significant differences in gaps closure between control and *GSN* KO cells only for the cells growing on laminin, Matrigel, and collagen type I (Figure 9). However, under conditions of laminin- and Matrigel-coatings, the cells without GSN overgrew the gap much slower when compared to control cells. On the contrary, in the case of collagen type I for the last 20 h, we noted faster gap closure for *GSN* KO clones than for control cells. Exemplary images acquired 72 h after starting the assay are shown in Supplementary Figure S11B.

Our results show that A375 cells migrate over longer distances on laminin and both types of collagen. A375 cells close the gap better on Matrigel but worse on laminin, collagens, and fibronectin when compared to non-coated surface. However, only for laminin-coating, we noted the decreased ability of the cells without GSN to migrate in both migration assays.

### 3.6. GSN-Depletion Heavily Impairs Invasion Abilities of A375 Cells

**Figure 10:**
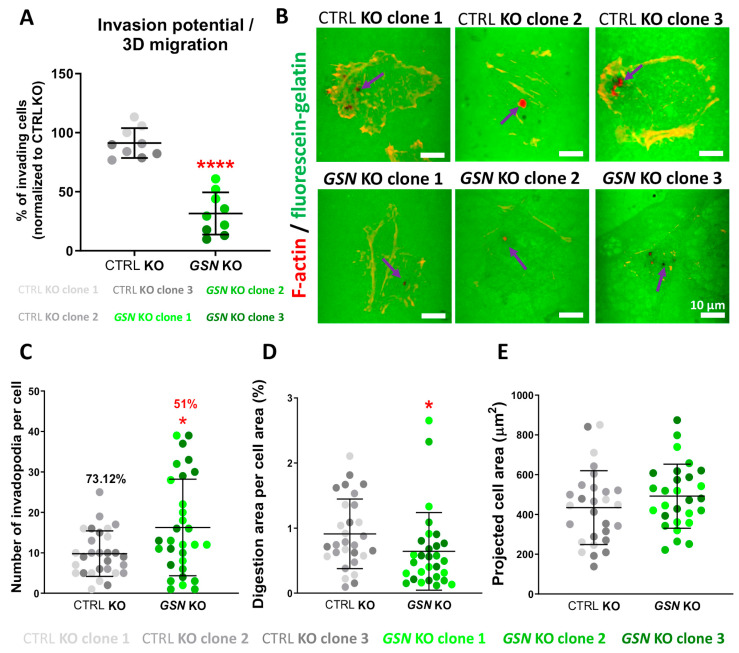
GSN-deprivation heavily affects A375 cells’ invasion. (**A**) Invasion assay (*n* = 9). (**B**) The ability of A375 clones to digest ECM was assessed with the fluorescein-gelatin assay. The cells were seeded onto gelatin-fluorescein-coated coverslips, and 24 h later, the cells were fixed and stained with fluorescently labeled phalloidin to detect F-actin. Violet arrows point at invadopodia with digestion potential. Dark spots represent cleaved fluorescent gelatin. Scale bar: 10 μm. (**C**) Number of invadopodia (the percentage above the bars in the graph refers to the percentage of active invadopodia co-localizing with the degradation area), (**D**) fluorescein-gelatin digestion area, and (**E**) projected cell area was calculated based on photos shown in B (*n* = 30). Results are expressed as the mean ± SD; *p* ≤ 0.05 (*) and *p* ≤ 0.0001 (****); unpaired t-test and Mann-Whitney test.

As we noticed that the 2-D migration abilities were lowered in the cells non-producing GSN, we decided to check the potential of the cells to migrate under 3-D conditions, in other words, to invade. For this purpose, we employed the invasion assay [34]. The cells, in order to migrate towards a chemoattractant, being here FBS, had to cross the Matrigel and then the pores of the transwell membrane. The number of *GSN* KO cells capable of invading dropped dramatically by about 60% in comparison to control cells (Figure 10A).

As MMPs activity is important for the mesenchymal mode of migration/invasion [29], and we showed that A375 cells migrate/invade in that way [34], we decided to check the gelatinase activity of studied here cells. The cells seeded on fluorescently labeled gelatin were additionally stained with phalloidin-Alexa Fluor 568 to detect F-actin. We noted that the *GSN* KO clones formed a higher number of invadopodia, which were less active. While 73% of detected invadopodia were digesting gelatin in control cells, only 51% of invadopodia formed by *GSN* KO cells had the potential to cleave gelatin (Figure 10B,C). The normalized per projected cell area (Figure 10E) digestion of fluorescently labeled gelatin was lower in comparison to control cells (Figure 10B,D).

Altogether, we show that GSN-depletion leads to heavy impairment of invasion abilities of A375 cells.

## 4. Discussion

Standard anti-tumor treatments, such as surgical resection, chemotherapy, and radiotherapy, are often ineffective in advanced metastatic melanoma, though the number of chemotherapeutics, including targeted chemotherapeutics, used to fight against melanoma is constantly growing [35]. Thus, precise knowledge about tumor cell dissemination is indispensable while designing new targeted drugs. Yet, a lot of remains to be discovered and understood in melanoma biology. Recently, more focus has been given to the role of the melanoma microenvironment in melanoma progression [36,37,38], including the acquisition of resistance to therapeutics [39]. For example, Hirata and colleagues showed that the growth of melanoma cells on the fibronectin-coated surface resulted in their blunted response to the PLX4720—BRAF V600D/E inhibitor [40]. The tumor niche comprises the ECM and depending on its composition, cell fate is regulated, determining cell migratory and adhesive properties [41].

Along with the progress of tumorigenesis, cells’ attachment to the BM is loosened. Mainly the alterations in the expression level of proteins involved in the cell’s adhesion are responsible for that [42,43]. However, the mechanisms lying at the core of cells’ behavior upon contact with ECM—a complicated network of macromolecules—are far from being understood. Here, we focused on the analysis of GSN’s impact on the formation of F-actin structures taking part in cells’ motility in response to the main fibrous proteins of the skin’s BM, i.e., laminin, collagen type IV, and fibronectin. We also evaluated several parameters connected with the motility of cells growing on collagen type I and Matrigel comprising mainly laminin and collagen type IV. We decided to look at melanoma cells’ behavior on selected ECM proteins because several studies reported changed BM composition during melanomagenesis [44,45]. For instance, it was shown that melanoma cells could degrade proteoglycans on the BM, altering its composition [46]. Whereas, Pasco et al. reviewed several matrikine peptides derived from BM proteins that can control melanoma progression by regulating the proteolytic cascade and promoting cell adhesion [47]. Additionally, it was discovered that the BM of patients with melanoma shows altered localization of laminin, which presence was noted only around the nodal nests of tumor cells but not in the deeper layers of the skin [44]. Moreover, the anchoring fibrils and collagen type VII are lost in the surrounding structures of the melanoma.

To study the role of GSN in melanoma cells’ motility on different ECM proteins, we decided to knockout *GSN*. For this purpose, we used the CRISPR/Cas9(D10A) system, which we utilized in our previous studies [25,31]. We believe that this was an appropriate approach because non-manipulated A375 cells exhibit a varying level of GSN. Moreover, we cannot exclude the possibility that the cells with low or no *GSN* expression are influenced by their neighbors with medium or high GSN levels via, e.g., secreted exosomes containing GSN. Recently, it was shown that melanoma exosomes contain GSN [48]. In future studies, it will be worth checking whether the exosomes of melanoma cell lines studied here comprise GSN. Interestingly, we found that not all copies of *GSN* in genomes of *GSN* KO clones had out-of-frame ORF. However, all copies of *GSN* were edited. We believe that no or very low (*GSN* KO clone no. 2) *GSN* expression in such a situation is a result of unstable mutated protein product being immediately degraded upon synthesis. Alternatively, it is possible that the *GSN* copy without out-of-frame ORF was not transcribed due to, e.g., acetylation, which is one of the mechanisms regulating *GSN* expression level in urinary bladder cancer cell lines [49]. Nevertheless, we want to stress here the fact that all *GSN* KO clones behaved in the same way, as they did not produce GSN. However, it is crucial to note here that the analyses of CRISPR/Cas9 gene editing should not be restricted solely to protein level evaluation. The parameters evaluated here, together with the changes in them caused by GSN-deprivation, are summarized in Table 1.

It has been reported that the rates of proliferation and migration affect cells’ turnover and, therefore, the rate of tumor evolution [50]. Under the conditions prevailing inside the tumor, when space is limited, cells switch to an increased rate of proliferation, as a result of which the rate of evolution of both migration and proliferation capacity increases. In contrast, the rate of migration is evolutionarily favored for cells at the edge of the tumor because it promotes cell spreading. As there is a link between migration and the proliferation potential of tumor cells, and because it was demonstrated that GSN promotes proliferation of human hepatocellular carcinoma [51], we decided to investigate the effect of various ECM proteins on melanoma cell proliferation, but we did not notice any effect on the cells’ proliferation upon GSN-deprivation. Instead, we noticed an A375 cell proliferation stimulating effect of collagen I, and this is consistent with the data presented by others [52].

Although we did not observe any significant changes in F-actin organization in non-manipulated A375 cells with varying GSN levels, we observed changes in the formation of F-actin-based structures and migratory capacity upon GSN-depletion. The most striking effects were seen for the laminin-coating. Already in 1999, it was reported that melanoma tumor microvessels were enriched in laminin β2, which according to the authors, promoted cell migration along the abluminal surface of vessels, thus stimulating melanoma cells invasion [53]. Moreover, it was found that melanoma appears to produce multiple isoforms of laminin and interact with them via integrin receptors [54] or 67 kDa non-integrin laminin receptor [55]. These interactions between melanoma cells’ receptors and laminins result in adhesion, migration, and metastasis propagation [56,57], which indicates a tumorigenic effect of laminins. Our results suggest the involvement of GSN in the interaction between laminin and melanoma cells since cells lacking *GSN* expression had a significantly impaired ability to migrate both spontaneously and collectively. We suspect that the involvement of GSN in the migration of melanoma cells on the laminin-coated surface may be related to the interaction of GSN with the non-integrin laminin receptor (LamR), which we have shown earlier [7]. Certainly, this issue requires further research to confirm our hypothesis.

Apparently, not only laminin is important for melanoma motility. Welf and colleagues have presented data showing that melanoma cells, while invading through matrix exhibited blebs, which at their perimeter mechanically destroyed collagen net [58]. Patients with melanoma characterized by a higher expression of the transcript for collagen I had lower median survival compared to the group of patients with lower collagen I transcript levels [52]. It has been shown that melanoma cells can stimulate and inhibit melanoma-associated fibroblasts in the context of collagen type I expression so that at various stages of melanoma progression, cells can employ their pro-angiogenic or pro-proliferative properties [59]. Moreover, it has been shown that highly invasive MV3 melanoma cells, through the action of integrin α2β1, can migrate through collagen type I gel and rebuild its lattices [60]. Collagen type IV was shown to promote adhesion, migration, and spreading of melanoma cells [61] via interaction with chondroitin sulfate proteoglycan via CD44 or β1 integrin [62,63] involving the signaling cascades related to p(Tyr127)FAK and paxillin [64], PI3-kinase, and protein kinase C (PKC) [65]. Lowering the level of *GSN* expression in leukocytes reduced the affinity of integrin β1 for collagen [66]. Perhaps as a result of depriving the melanoma cells of GSN synthesis, the interaction of β1 integrin with collagens type I and IV was disturbed, which influenced the ability of the studied cells to migrate on the surface coated with collagens. Correlation has been demonstrated between high levels of collagen type I and fibronectin expression in melanoma, as well as an increased level of fibronectin in the ECM surrounding melanoma cells [52]. The interaction of α5 integrin with fibronectin is important for the metastasis of murine B16F10 melanoma cells and their escape from apoptosis [67]. Fibronectin is a chemoattractant that stimulates the migration of melanoma cells [63], and reducing its expression lowers the ability of melanoma cells to migrate [68]. However, we did not observe an effect for this protein on the migration of melanoma cells that were moving across a fibronectin-coated surface.

An important observation here is that filopodia formation was heavily influenced by GSN-depletion. It was previously reported that filopodia are important for tumor cells as their formation is correlated with metastasis [69]. Strikingly, under laminin-coating conditions, GSN-depletion resulted in a higher number of filopodia, their increased length, and their density when compared to control cells. The better filopodial activity was inversely correlated with *GSN* KO cells’ motility on laminin. It appears that if filopodia adhere to the substrate to form focal complexes, cell movement may be restricted [70,71]. Interestingly, the hippocampus cells of *GSN* knockout mice were shown to form significantly more filopodia along the neurites compared to the control. The analysis of individual filopodia showed that they do not differ in elongation rate, but as a result of the loss of GSN synthesis, they have a disturbing retraction phase [72]. Another study confirmed these observations by showing that overexpression of GSN in human esophageal cancer cells resulted in a reduction in the number of filopodia [73]. Thus, the reduced migration capacity of GSN-deprived melanoma cells on the laminin-coated surface may be associated with impaired filopodia retraction. On the other hand, the *GSN KO* cells formed less thick actin bundles on laminin. One could speculate that the adhesive properties of these cells could be thus altered, ultimately affecting cells’ migratory potential. This hypothesis is worth evaluation in future studies. Recently, Li with colleagues showed that filopodial activity is important for cell spreading and adhesion on fibronectin and galectin-8 [74]. However, they noticed that the cells spreading over galectin-8 formed a higher number of filopodia (but no stress fibers and focal adhesions) in comparison to the cells cultured on fibronectin. Moreover, the cells adhered to galectin-8 with a higher force than to fibronectin. Adhesion has a twofold effect on the mobility of cells as they migrate fast when growing in the presence of medium concentrations of ECM proteins, i.e., moderate adhesion strength, and slowly with high or low concentrations of ECM proteins, i.e., strong or weak adhesion strengths. These observations can be explained by the fact that too weak adhesion does not provide sufficient support to move, while too strong adhesion causes cell immobilization. The balance between migration and adhesion is maintained through the interactions between actin, myosin, and focal adhesion formation dynamics [75]. Interestingly, we observed that the projected cell area of A375 cells is different depending on the ECM protein. This implies that the composition or mechanics of the cell’s adhesive structures is different as a response to selected ECM fibrous proteins. As we observed differences between control and *GSN* KO cells regarding the projected cell area of A375 cells, we assume that there are some alterations in cell adhesion, e.g., force, as their motility features investigated here are changed mainly for laminin but also collagens. Additionally, it is crucial to note here that the differences in the projected cell area depending on the coating together with the enhanced proliferation of the cells growing on collagen type I are reflected in the diverse confluency of the A375 growing on studied here ECM proteins. While the higher confluency was observed for the cells growing on collagens and fibronectin when compared to control cells, lower confluency was noted for laminin. There are very scant data on GSN’s role in adhesion, though it was shown that GSN is involved in adhesion. GSN was found to be crucial for inside-out control of β1 integrin in leukocytes by triggering conformational changes in the β1 integrin moiety [76] and for activation of β1 integrin, and thus cell adhesion of mouse acute lymphocytic leukemia cells [66]. However, the mechanism of GSN’s action on cells’ adhesion remains to be elucidated.

We have previously shown that GSN resides in invadopodia of melanoma cells [7], where it interacts with Arp3, a known invadopodium constituent [77]. In this study, we corroborate this observation, and we show that GSN occupies the same F-actin-rich spots, which are cortactin-positive. Interestingly, it was recently proven that GSN interacts with cortactin in human pancreatic ductal adenocarcinoma cells (PDAC) [78], though the authors showed GSN co-localization with cortactin at the cell periphery and not in invadopodium. It was noted that in PDAC cells, the levels of GSN and cortactin were increased in spheroids when compared to the monolayer implicating the important role of GSN in migration and invasion of those cells. Here, we did not observe any dramatic changes in invadopodia formation in cells devoid of GSN. When the cells were growing on a non-coated surface, they formed surprisingly more invadopodia than control cells. However, the results from the fluorescein-gelatin assay showed that the cells without GSN, which manifested heavily impaired invasion/3-D migration abilities, formed a higher number of invadopodia, albeit they were less active than in the case of control cells. A higher number of invadopodia was previously observed by us when we studied A375 cells not producing β or γ actin or actbl2 [25,31]. We assume that this phenomenon is a somewhat compensation mechanism activated by those cells with diminished migratory abilities. Gelatin is a denatured mixture of collagens [79]. Taking into account the outcomes for invadopodia formation assessed separately for collagens type I and IV, it indicates that the effects observed for complex ECM compositions cannot be directly extrapolated from the observations done for a single ECM protein. This was also seen in the study on cells’ spreading and adhesion properties on galectin-8 and fibronectin and their combinations at different ratios [74]. Summarizing this section, it is crucial to state that GSN is apparently important for the proper functioning of invadopodia as it resides there.

Finally, we looked at the invasion potential of GSN-deprived cells. We noted a greater than 60% fall in the number of cells able to migrate in 3-D conditions in the presence of gel resembling the skin’s BM. Earlier, we observed impaired migration of A375 cells upon application of siRNA targeting GSN’s mRNA [6]. However, in the former study, we did not use Matrigel. The cells had to solely cross the transwell’s membrane barrier towards a chemoattractant. Thus, we show here for the first time that GSN is crucial for A375 melanoma cells invasion.

It is also important to mention here the issue of phenotypic heterogeneity concerning GSN level in melanoma cell lines, which we noticed in this study. Recently, we noted that A375 cells differ in their response to knockdown of *TMSB4X* expression, the gene coding for thymosin β4, an actin sequestering protein, which pointed at the phenotypic heterogeneity of A375 cells [34]. This issue was discussed extensively in that paper. Thus, we will not repeat this here. However, we want to highlight the intriguing fact that we observed for the control clones, which originated from single cells, restoration of the distribution ratio of GSN level within their populations. This suggests a strict control of *GSN* expression in these cells and the importance of preservation of particular distribution ratio of the cells with varying GSN levels. This is certainly an important issue that should be investigated as part of future work.

## 5. Conclusions

To study the effects of ECM proteins is of high importance. In their splendid review on the integrin signaling in cancer [80], Cooper and Giancotti recently stated that our understanding of the interplay between remodeled tumor ECM and signaling into the cell via deregulated integrins, major ECM receptors, remains rather poor. They stress that alterations in the expression of ECM proteins and integrins, together with changes in tissue stiffness, can lead to the dissemination of tumor cells, enabling them to find new niches to inhabit. Thus, we believe that our study gives new important knowledge about the role of GSN in melanoma cell migration, which is dependent on cells’ attachment to the ECM mediated mainly by integrins [81], but also by other types of ECM receptors, such as discoidin domain receptors and cell surface proteoglycans [82]. In conclusion, we show here that A375 cells’ motility on laminin is dependent on GSN. Moreover, this protein is crucial for the invasion of melanoma cells. As the next step, we plan to investigate the adhesion of *GSN* KO to ECM proteins to determine the link between motility and adhesion in the context of GSN’s action.

## Figures and Tables

**Table 1:** Summary of the effects observed for the *GSN* KO cells in comparison to control cells depending on the type of coating. The blue rectangle represents no changes, while green and red arrows represent a rise or fall, respectively.

	Coating	No Coating	Laminin	Fibronectin	Collagen Type I	Collagen Type IV	Matrigel
Feature							
Proliferation		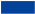	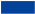	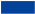	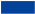	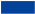	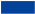
Projected cell area		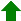	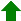	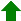	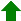	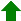	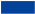
Number of filopodia		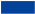	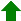	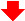	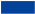	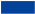	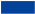
Number of filopodia		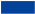	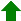	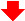	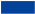	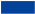	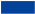
Length of filopodia		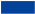	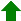	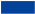	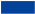	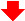	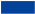
Spontaneous migration		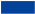	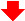	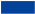	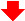	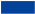	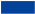
Collective migration		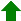	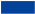	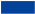	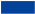	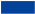	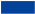
Spontaneous migration		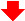	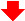	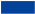	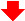	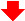	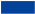
Collective migration		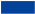	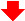	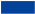	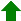	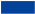	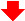
		**Mixed Coatings**					
Active invadopodia		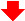					
Invasion potential		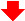					

## Data Availability

Not applicable.

## Supplementary Materials

The following are available online at https://www.mdpi.com/article/10.3390/cells10081848/s1, Figure S1: The way the distribution ratio of the cells with different GSN level was calculated. Figure S2: Verification of cell clones’ correctness. Figure S3: GSN is produced in *GSN* KO clone 2 at a very low level. Figure S4: Analysis of gDNA of obtained clones to check whether gene editing took place. Figure S5: Among *GSN* KO clones, only *GSN* KO clone 2 produces small amounts of GSN. Figure S6: Some ECM proteins have an influence on A375 cells proliferation. Figure S7: ECM proteins have an impact on A375 cells’ confluence. Figure S8: Different influence of ECM proteins on the A375 projected cell’s area. Figure S9: Evaluation of the impact of different ECM proteins on the formation of filopodia, thick actin bundles and invadopodia in control and *GSN* KO clones. Figure S10: ECM proteins differently influence spontaneous migration of control and *GSN* KO clones. Figure S11: Depending on the ECM protein there are differences in collective migration of control and *GSN* KO clones.

## Abbreviations

BM	basement membrane
CTRL KO	control cells
*GSN* KO	cells with knocked out GSN
CRISPR	Clustered Regularly Interspaced Short Palindromic Repeats
ECM	extracellular matrix
F-actin	filamentous actin
FBS	fetal bovine serum
gDNA	genomic DNA
*GSN*	gene coding for gelsolin
GSN	gelsolin
MMPs	metalloproteases
SIM	structured illuminated microscopy
