# Supplementary material for: Gelsolin Contributes to the Motility of A375 Melanoma Cells and This Activity Is Mediated by the Fibrous Extracellular Matrix Protein Profile
Source: Cells. 2021 Jul 21;10(8):1848. doi: 10.3390/cells10081848 (PMC8394273; doi:10.3390/cells10081848)
Supplement: Supplementary file 1 [file cells-10-01848-s001.zip › cells-1267028-supplementary.pdf]

Supplementary Material

Gelsolin contributes to the motility of A375 melanoma cells and this activity is mediated by the fibrous extracellular matrix protein profile

Ewa Mazurkiewicz, Aleksandra Makowiecka, Ewa Mrówczyńska, Iryna Kopernyk, Dorota Nowak, Antonina J. Mazur*

Department of Cell Pathology, Faculty of Biotechnology, University of Wroclaw, Poland

***Corresponding author:**
Dr. Antonina Joanna Mazur, DSc
antonina.mazur@uwr.edu.pl
Department of Cell Pathology
Faculty of Biotechnology
University of Wroclaw
ul. Joliot-Curie 14a
50-383 Wrocław
tel. + 48 71 37 56 206

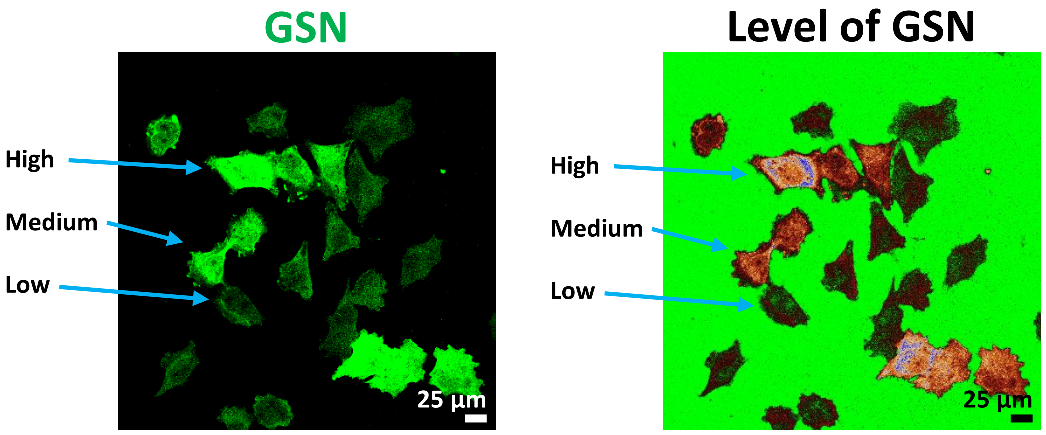

Fig. S1 The way the distribution ratio of the cells with different GSN level was calculated. The „Over-/Underexposure" tool of the LasX software (Leica) was used to assess the intensity of fluorescence signal in the A375 cells and control clones. This figure corresponds to Fig. 2B and 3C in the main text. Scale bar: 25 µm.

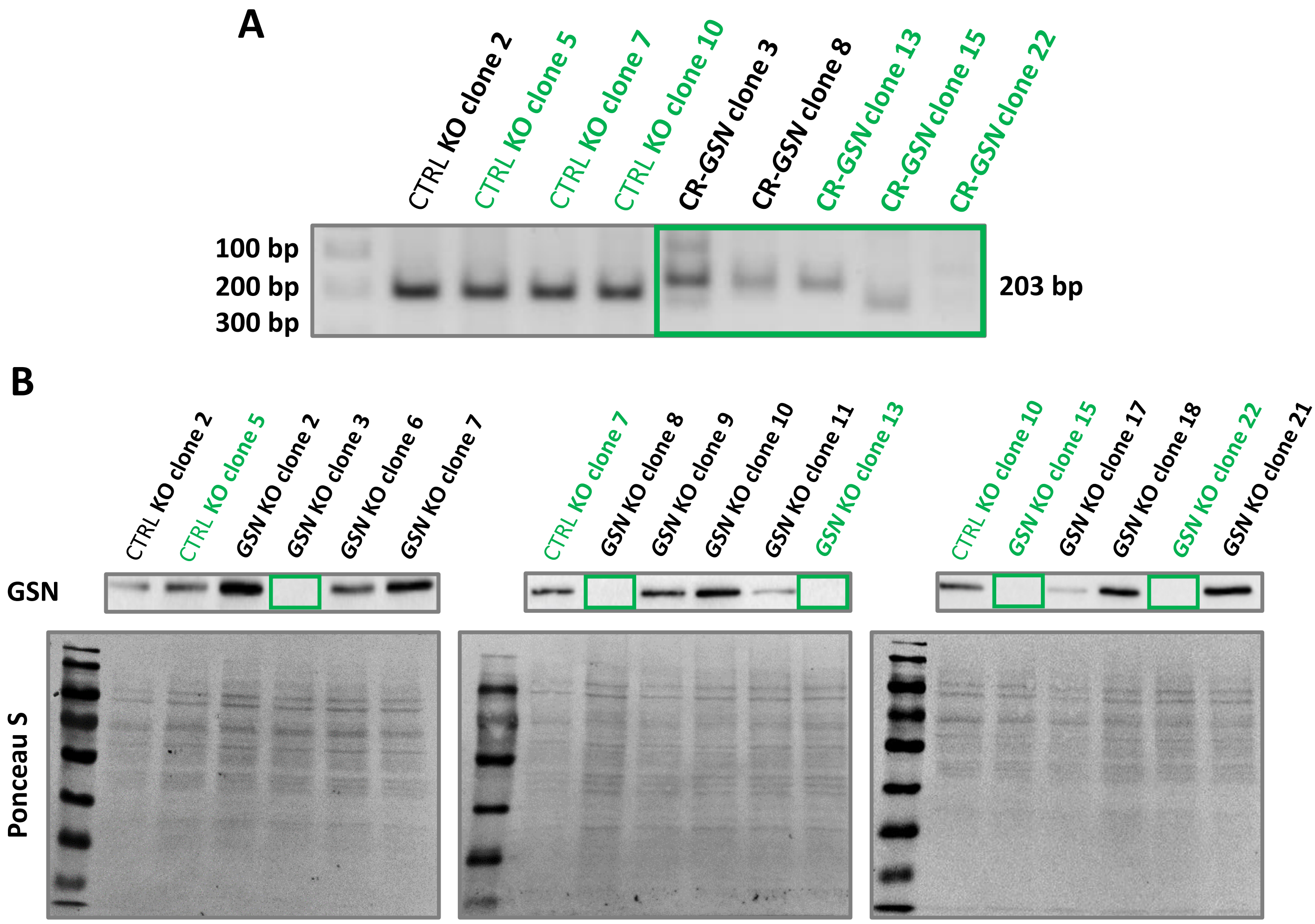

C

The initial number of the clone	The final number of the clone
CTRL **KO clone 5**	CTRL **KO clone 1**
CTRL **KO clone 7**	CTRL **KO clone 2**
CTRL **KO clone 10**	CTRL **KO clone 3**
GSN **KO clone 13**	***GSN*** **KO clone 1**
GSN **KO clone 15**	***GSN*** **KO clone 2**
GSN **KO clone 22**	***GSN*** **KO clone 3**

Fig. S2 Verification of cell clones' correctness. (**A**) Analysis of gDNA of A375 cell clones obtained upon the usage of CRISPR/Cas9(D10A) technique. 100 ng of isolated gDNAs served as templates for subsequent PCR reactions performed with appropriate starters. Products of PCR reactions were analyzed in 2% TAE agarose gel. bp - base pairs. (**B**) Western blot analysis of clones' lysates for the presence of GSN. 30 µg of protein were loaded on every lane. Ponceau S membrane stainings were performed as total protein analysis (TPA) of the membranes. (**C**) Nomenclature of the clones used in the study.

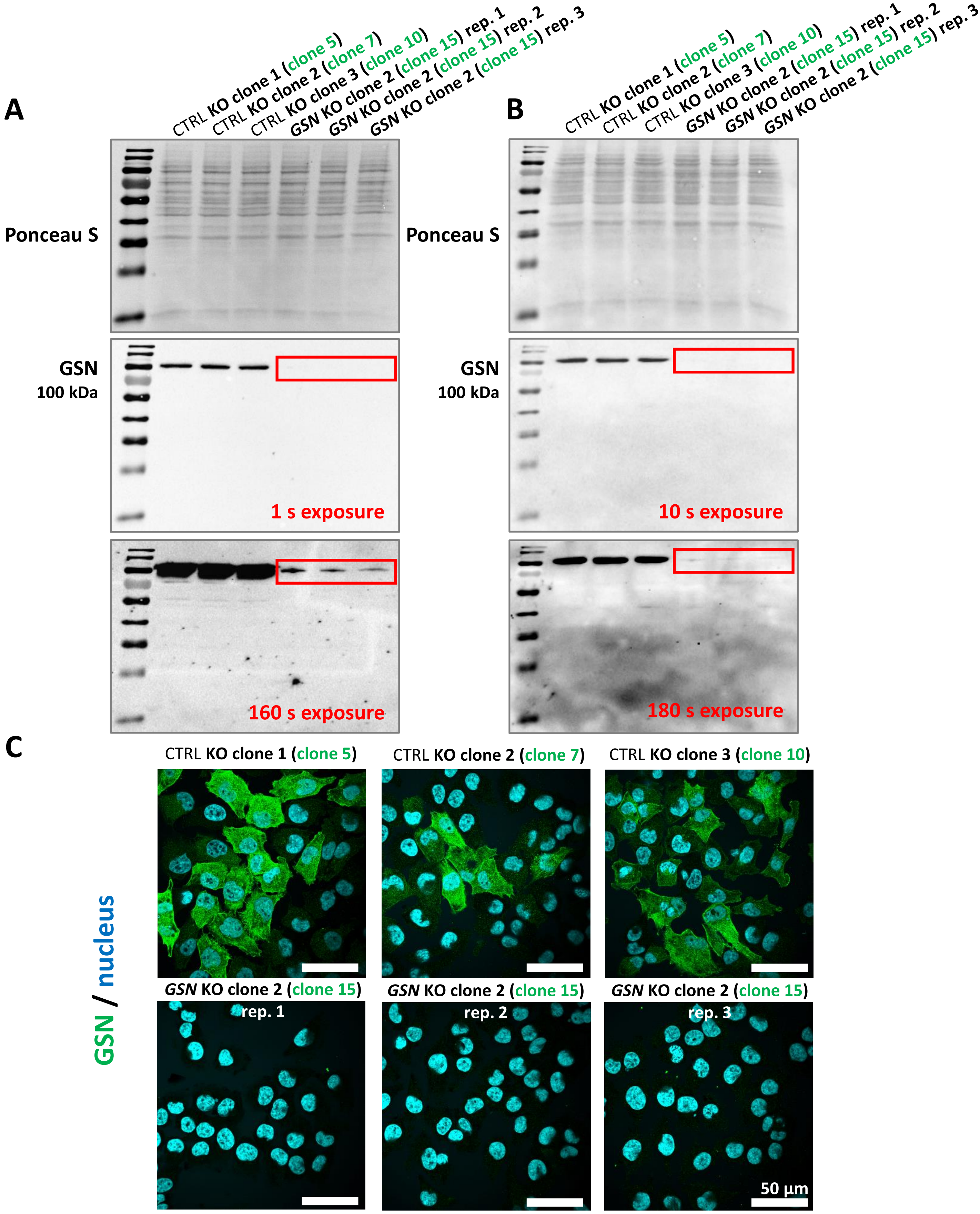

Fig. S3 GSN is produced in *GSN* KO clone 2 at a very low level. Western blot analysis of clones' lysates for the presence of GSN. The membranes were probed for GSN with mouse anti-GSN (**A**) and goat anti-GSN (**B**) antibodies. 30 µg of protein were loaded on every lane. Long and short exposure times were applied. Red rectangles highlight GSN bands in three times collected lysates of *GSN* KO clone 2. Ponceau S membrane stainings were performed as total protein analysis (TPA) of the membranes. (**C**) Immunocytochemical analysis of three control clones and three repetitions of *GSN* KO clone 2. GSN was detected by application of mouse anti-GSN antibodies, whereas F-actin and nucleus were detected by using fluorescently labeled phalloidin and Hoechst 33342. Staining for *GSN* KO clone 2 was repeated three times. Images were acquired at the same settings. Scale bar: 50 µm.

```
CTRL_KO          GGCTTTCTCAGCTTCTACAGCTAGGTCAGGGCAGTGCTGGGGTTCCTCCTCCACCTCCTC
GSN_KO_cl1.1     GGCTTTCTCAGCTTCTACAGCTAGGTCAGGGCAGTGCTGGGGTTCCTCCTCCACCTCCTC
GSN_KO_cl1.2     GGCTTTCTCAGCTTCTACAGCTAGGTCAGGGCAGTGCTGGGGTTCCTCCTCCACCTCCTC
GSN_KO_cl2.1     GGCTTTCTCAGCTTCTACAGCTAGGTCAGGGCAGTGCTGGGGTTCCTCCTCCACCTCCTC
GSN_KO_cl2.2     GGCTTTCTCAGCTTCTACAGCTAGGTCAGGGCAGTGCTGGGGTTCCTCCTCCACCTCCTC
GSN_KO_cl3.1     GGCTTTCTCAGCTTCTACAGCTAGGTCAGGGCAGTGCTGGGGTTCCTCCTCCACCTCCTC
GSN_KO_cl3.2     GGCTTTCTCAGCTTCTACAGCTAGGTCAGGGCAGTGCTGGGGTTCCTCCTCCACCTCCTC
                 ************************************************************

CTRL_KO          TTCCTCAGGGGTCTGGGATACTTCTG--GAAAGCCAGGCTCATATTGCTCTGATGTC---
GSN_KO_cl1.1     TTCCTCAGGGGTCTGGGATACTTCTG--GAAAGCCAGGCTCATATTGCTCTGATGTC---
GSN_KO_cl1.2     TTCCTCAGGGGTCTGGGATACTTCTG--GAAAGCCAGGCTCATATTGCTCTGATGTC---
GSN_KO_cl2.1     TTCCTCAGGGGTCTGGGATACTTCTG--GAAAGCCAGGCTCATATTGCTCTGATGTC---
GSN_KO_cl2.2     TTCCTCAGGGGTCTGGGATACTTCTG--GAAAGCCAGGCTCATATTGCTCTGATGTC---
GSN_KO_cl3.1     TTCCTCAGGTGCAGCTGGATGACTACCTGAACGGCCGGGCCGTGTTACCGTGCAGCTGGA
GSN_KO_cl3.2     TTCCTCAGGGGTCTGGGATACTTCTG--GAAAGCCAGGCTCATATTGCTCTGATGTC---
                 ********* *     *           *** * * **  * * ** *  **  *

CTRL_KO          ------------------------------CCGTAGG-----------------------
GSN_KO_cl1.1     ------------------------------CCGTAGG-----------------------
GSN_KO_cl1.2     ------------------------------CCGTAGG-----------------------
GSN_KO_cl2.1     ------------------------------CCGTAGG-----------------------
GSN_KO_cl2.2     ------------------------------CCGTAGG-----------------------
GSN_KO_cl3.1     TGACTACCTGAACGGCCGGGCCGTGCAGCACCGTGCAGCTGGATGACTACCTGAACGGCC
GSN_KO_cl3.2     ------------------------------CCGTAGG-----------------------
                                               ****

CTRL_KO           ---CA----ATGAGTGCAGCCAG-------------------------------------
GSN_KO__cl1.1     ---CA----ATGAGTGCAGCCAG-------------------------------------
GSN_KO__cl1.2     ---CA----ATGAGTGCAGCCAG-------------------------------------
GSN_KO__cl2.1     ---CA----ATGAGTGCAGCCAG-------------------------------------
GSN_KO__cl2.2     ---CA----ATGAGTGCAGCCAG-------------------------------------
GSN_KO__cl3.1     GGGCCGTGTTACCGTGCAGCTGGATGACTACCTGAACGGCCGGGCCGTGCAGCACCGTGC
GSN_KO__cl3.2     ---CA----ATGAGTGCAGCCAG-------------------------------------
                    *         *******  *

CTRL_KO          -----GATGA-GAGCGGGGC-GGCCGCCATCTTTACCGTGC-------------------
GSN_KO_cl1.1     -----GATGA-GAGCGGGGC-GGCCGCCATCTTTACCGTGCAGCTG--------------
GSN_KO_cl1.2     -----GATGA-GAGCGGGGC-GGCCGCCATCTTTACCGTGCAGCTG--------------
GSN_KO_cl2.1     -----GATGA-GAGCGGGGC-GGCCGCCATCTTTACCGTGCAGCTG--------------
GSN_KO_cl2.2     -----GATGA-GAGCGGGGC-GGCCGCCATCTTTACCGTGCAGCTG--------------
GSN_KO_cl3.1     AGCTGGATGACTACCTGAACGGCCGGGCCGTGTTACCGTGCAGCTGGATGACTACCTGAA
GSN_KO_cl3.2     -----GATGA-GAGCGGGGC-GGCCGCCATCTTTACCGTGC-INSERTION-1117nt--
                      *****  * * *  * * * * *    *********

CTRL_KO          ---------------------------------------------------------AGC
GSN_KO_cl1.1     -----------------------------GAT------------------------GACT
GSN_KO_cl1.2     -----------------------------GAT------------------------TTAC
GSN_KO_cl2.1     -----------------------------AAT------------------------GACT
GSN_KO_cl2.2     -----------------------------GAT------------------------GAGC
GSN_KO_cl3.1     CGGCCGGGCCGTGCAGCACCGTGCAGCTGGATGACTACCTGAACGGCCGGGCCGTGTTAC
GSN_KO_cl3.2     ---------------------------------------------------------AGC

CTRL_KO          TGGATGA-----------------------------------------------------
GSN_KO_cl1.1     ACCTGAAC-------------------------------------------GGCCGGGCC
GSN_KO_cl1.2     CGTGCAGC-------------------------------------------TGGATGACT
GSN_KO_cl2.1     ACC---------------------------------------------------------
GSN_KO_cl2.2     TCACGGGC----------------------------------------------------
GSN_KO_cl3.1     CGTGCAGCTGGATGACTACCTGAACGGCCGGGCCGTGCAGCACCGTGCAGCTGGATGACT
GSN_KO_cl3.2     TGGATGA-----------------------------------------------------

CTRL_KO          -----------------------CTAC----------------CTGAACGGCCGGGCCGT
GSN_KO_cl1.1     GTG-----------C------AGCACC----------------TATGACTACCTGAACGT
GSN_KO_cl1.2     ACCTGAACGGCCGGT------TACCGT----------------GCAGCTGGATTTACCGT
GSN_KO_cl2.1     ------------------------------------------------------------
GSN_KO_cl2.2     ------------------------------------------------------------
GSN_KO_cl3.1     ACCTGAACGGCCGGGCCGTGTTACCGTGCAGCTGGATGACTACCTGAACGGCCGGGCCGT
GSN_KO_cl3.2     -----------------------CTAC----------------CTGAACGGCCGGGCCGT

CTRL_KO          GCAGCACCGTGAGGTCCAGGGCTTCGAGTCGGCCACCTTCCTAGGCTACTTCAAGTCTGG
GSN_KO_cl1.1     GCAGCACCGTGAGGTCCAGGGCTTCGAGTCGGCCACCTTCCTAGGCTACTTCAAGTCTGG
GSN_KO_cl1.2     GCAGCTGGATGAGGTCCAGGGCTTCGAGTCGGCCACCTTCCTAGGCTTCTTCAAGTCTGG
GSN_KO_cl2.1     ---TGATGGTGAGGTCCAGGGCTTCGAGTCGGCCACCTTCCTAGGCTACTTCAAGTCTGG
GSN_KO_cl2.2     -CGTGCAGCACCGGTCCAGGGCTTCGAGTCGGCCACCTTCCTAGGCTACTTCAAGTCTGG
GSN_KO_cl3.1     GCAGCACCGTGAGGTCCAGGGCTTCGAGTCGGCCACCTTCCTAGGCTACTTCAAGTCTGG
GSN_KO_cl3.2     GCAGCACCGTGAGGTCCAGGGCTTCGAGTCGGCCACCTTCCTAGGCTACTTCAAGTCTGG
                             *********************************** ************

CTRL_KO          CCTGAAGTACAAGGTGGGTTGGGCCCCACCTTGCTTGAGCGGTAGGGACAGATGCACCAG
GSN_KO_cl1.1     CCTGAAGTACAAGGTGGGTTGGGCCCCACCTTGCTTGAGCGGTAGGGACAGATGCACCAG
GSN_KO_cl1.2     CCTGAAGTACAAGGTGGGTTGGGCCCCACCTTGCTTGAGCGGTAGGGACAGATGCACCAG
GSN_KO_cl2.1     CCTGAAGTACAAGGTGGGTTGGGCCCCACCTTGCTTGAGCGGTAGGGACAGATGCACCAG
GSN_KO_cl2.2     CCTGAAGTACAAGGTGGGTTGGGCCCCACCTTGCTTGAGCGGTAGGGACAGATGCACCAG
GSN_KO_cl3.1     CCTGAAGTACAAGGTGGGTTGGGCCCCACCTTGCTTGAGCGGTAGGGACAGATGCACCAG
GSN_KO_cl3.2     CCTGAAGTACAAGGTGGGTTGGGCCCCACCTTGCTTGAGCGGTAGGGACAGATGCACCAG
                 ************************************************************
```

Fig. S4 Analysis of gDNA of obtained clones to check whether gene editing took place. Upon isolation of gDNA from the clones, PCR reactions were performed with appropriate starters and gDNAs as templates. Products were then cloned into the pAcGFP-C1 plasmid and selected plasmid clones were sequenced. The alleles with changes in the coding regions but without shifted ORFs are highlighted in red. The other alleles have out of frame open reading frame (ORF) in the region coding for GSN. The star indicates identical nucleotide in a given position for every allele.

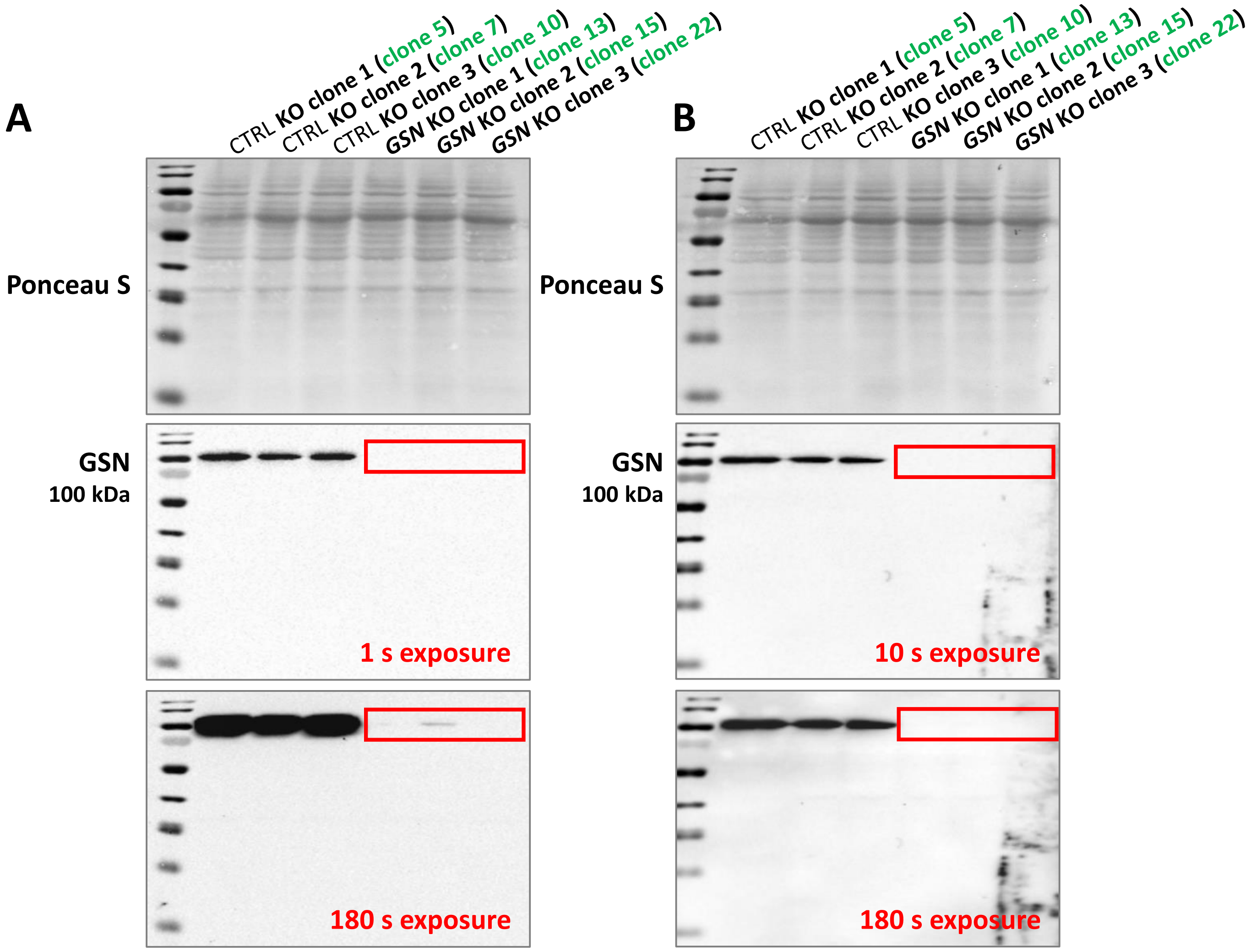

Fig. S5 Among *GSN* KO clones, only *GSN* KO clone 2 produces small amounts of GSN. Western blot analysis of clones' lysates for the presence of GSN. The membranes were probed for GSN with mouse anti-GSN (**A**) and goat anti-GSN (**B**) antibodies. 30 µg of protein were loaded on every lane. Long and short exposure times were applied. Red rectangles highlight GSN bands in the lysates of *GSN* KO clones. Ponceau S membrane stainings were performed as total protein analysis (TPA) of the membranes.

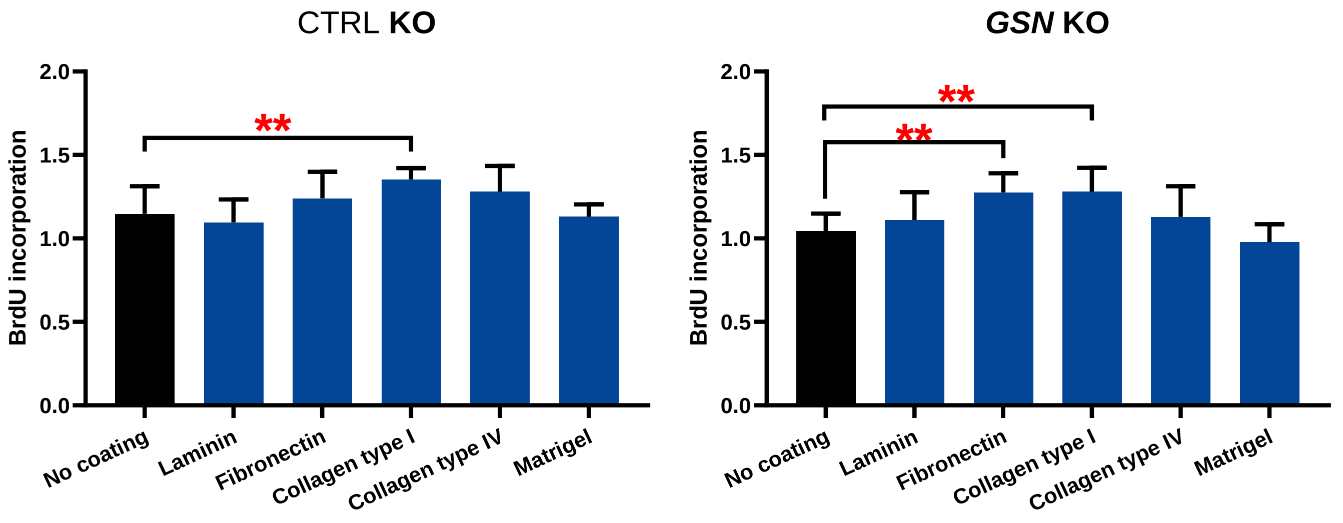

Fig. S6 Some ECM proteins have an influence on A375 cells proliferation rate. BrdU assay was performed on cells growing on different ECM proteins for 72 h (n = 8-9); Kruskal-Wallis one-way ANOVA test with post hoc (Dunn's multiple comparisons) test. Results are expressed as the mean ± SD; $p \leq 0.01$ (**). This figure corresponds to Fig. 5A in the main text. The results were calculated separately for CTRL KO and *GSN* KO clones.

A

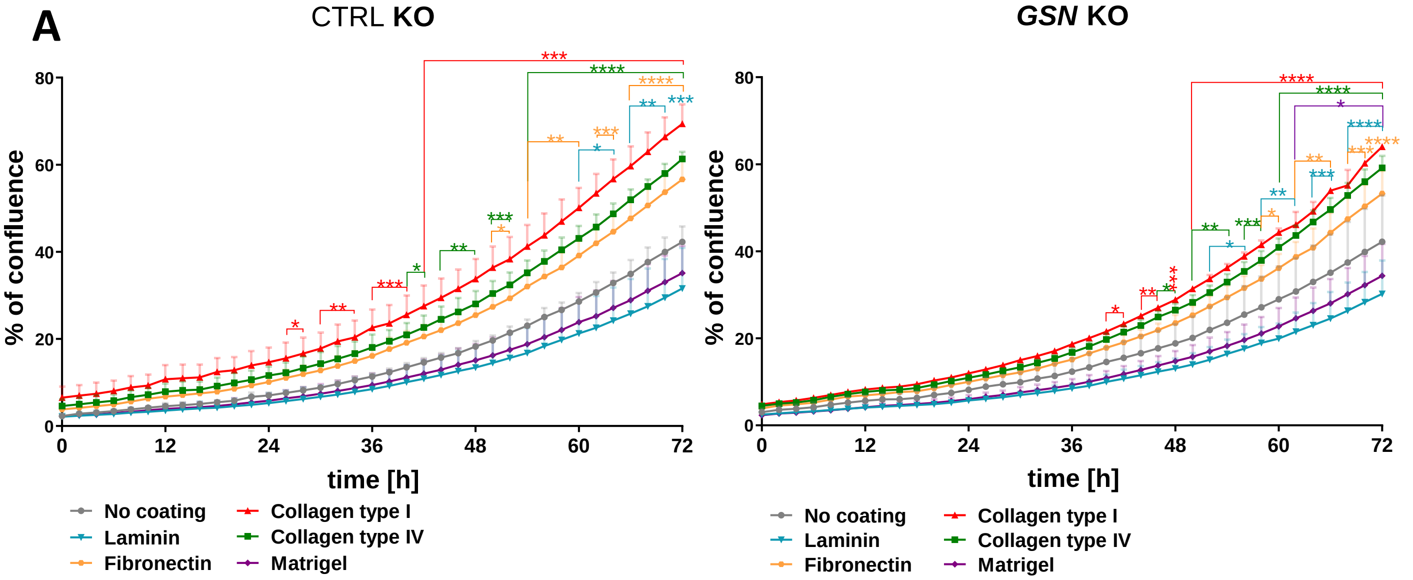

CTRL KO					
	Laminin	Fibronectin	Collagen I	Collagen IV	Matrigel
*	60-64 h	50-52 h	26-28 h	40-42 h	-
**	66-70 h	54-60 h	30-34 h	44-48 h	-
***	72 h	62-64 h	36-40 h	50-52 h	-
****	-	66-72 h	42-72 h	54-72 h	-

GSN KO					
	Laminin	Fibronectin	Collagen I	Collagen IV	Matrigel
*	52-56 h	58-60	40-42 h	46-48 h	62-72 h
**	58-62 h	62-66	44-46 h	50-54 h	-
***	64-66 h	68-70	48 h	56-58 h	-
****	68-72 h	72	50-72 h	60-72 h	-

B

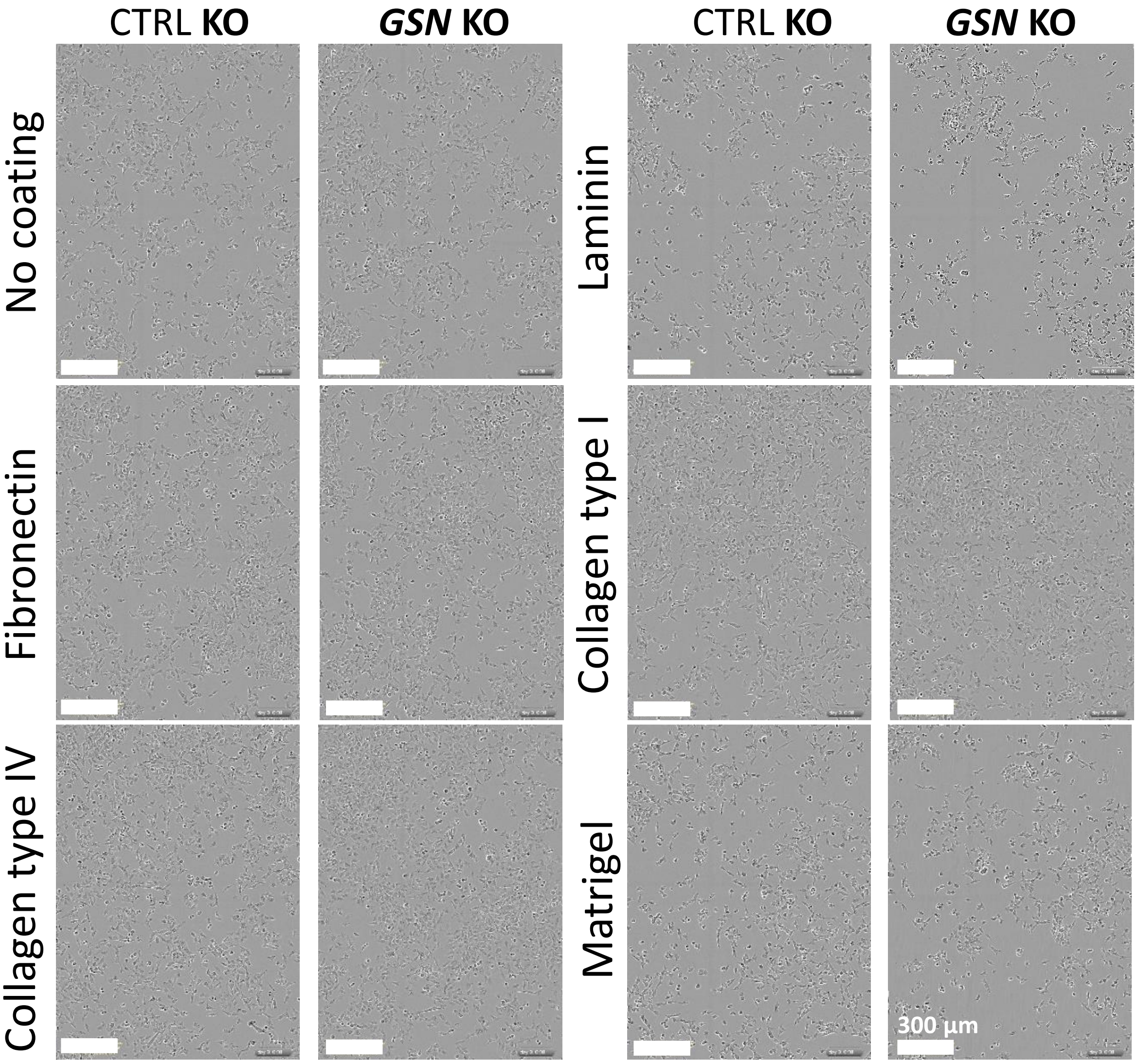

Fig. S7 ECM proteins have an impact on A375 cells' confluence. (**A**) Confluence was assayed with the help of the IncuCyte system. The cells seeded into coated wells of 96-well plate were photographed every 2 h over 72 h. Next, the confluence was calculated (n = 3). Statistical significance is shown both on the graphs and in a table; $p \leq 0.01$ (*), $p \leq 0.01$ (**), $p \leq 0.001$ (***) and $p \leq 0.0001$ (****); two-way ANOVA with post hoc (Dunn's multiple comparisons) test. The results were calculated separately for the CTRL KO and *GSN* KO clones. (**B**) Representative photos taken 72 h after seeding the cells onto studied here ECM proteins. This figure corresponds to Fig. 5B in the main text.

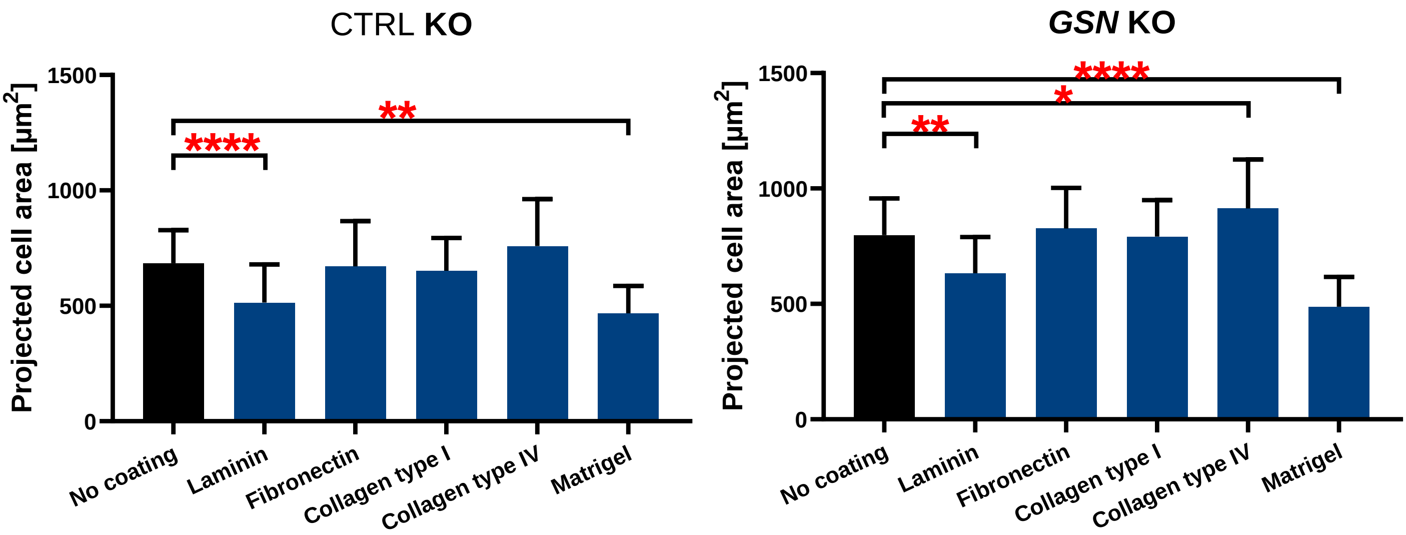

Fig. S8 Different influence of ECM proteins on the A375 projected cell's area. On the basis of the photos taken 48 h after seeding the cells onto coated coverslips with selected ECM proteins (presented in Fig. 7) the projected cell's area was evaluated with ImageJ as described in the materials and methods section (n = 30); Kruskal-Wallis one-way ANOVA test with post hoc (Dunn's multiple comparisons) test. Results are expressed as the mean ± SD; $p \leq 0.05$ (*), $p \leq 0.01$ (**) and $p \leq 0.0001$ (****). This figure corresponds to Fig. 6 in the main text. The results were calculated separately for the CTRL KO and *GSN* KO clones.

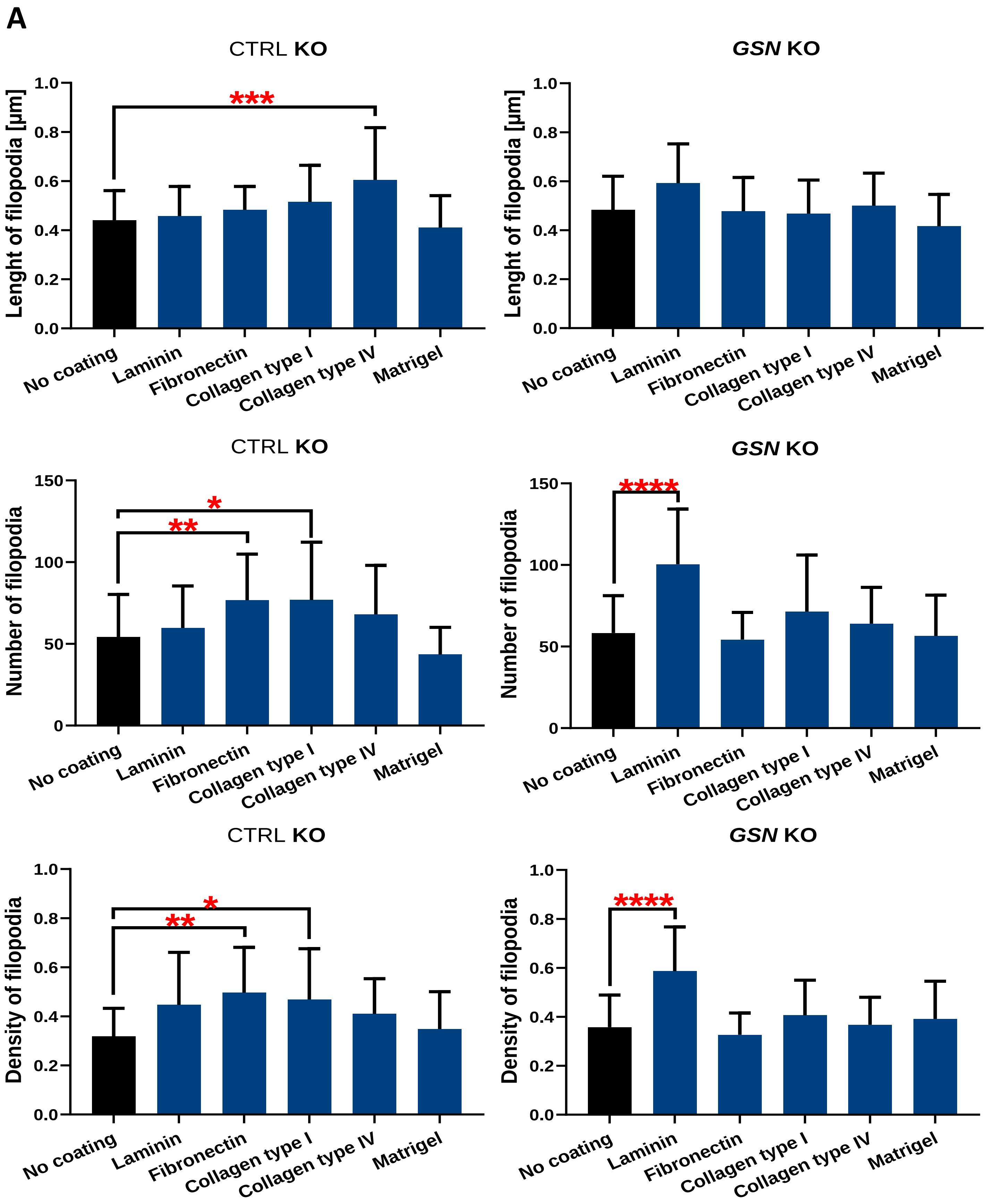

To be continued

B

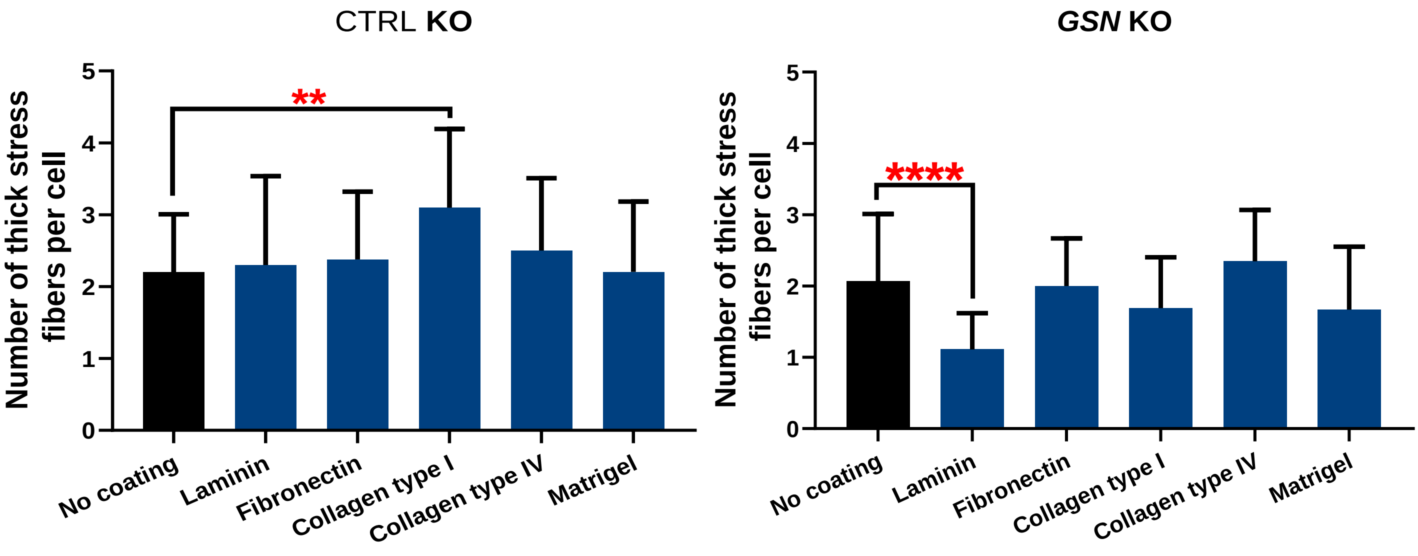

C

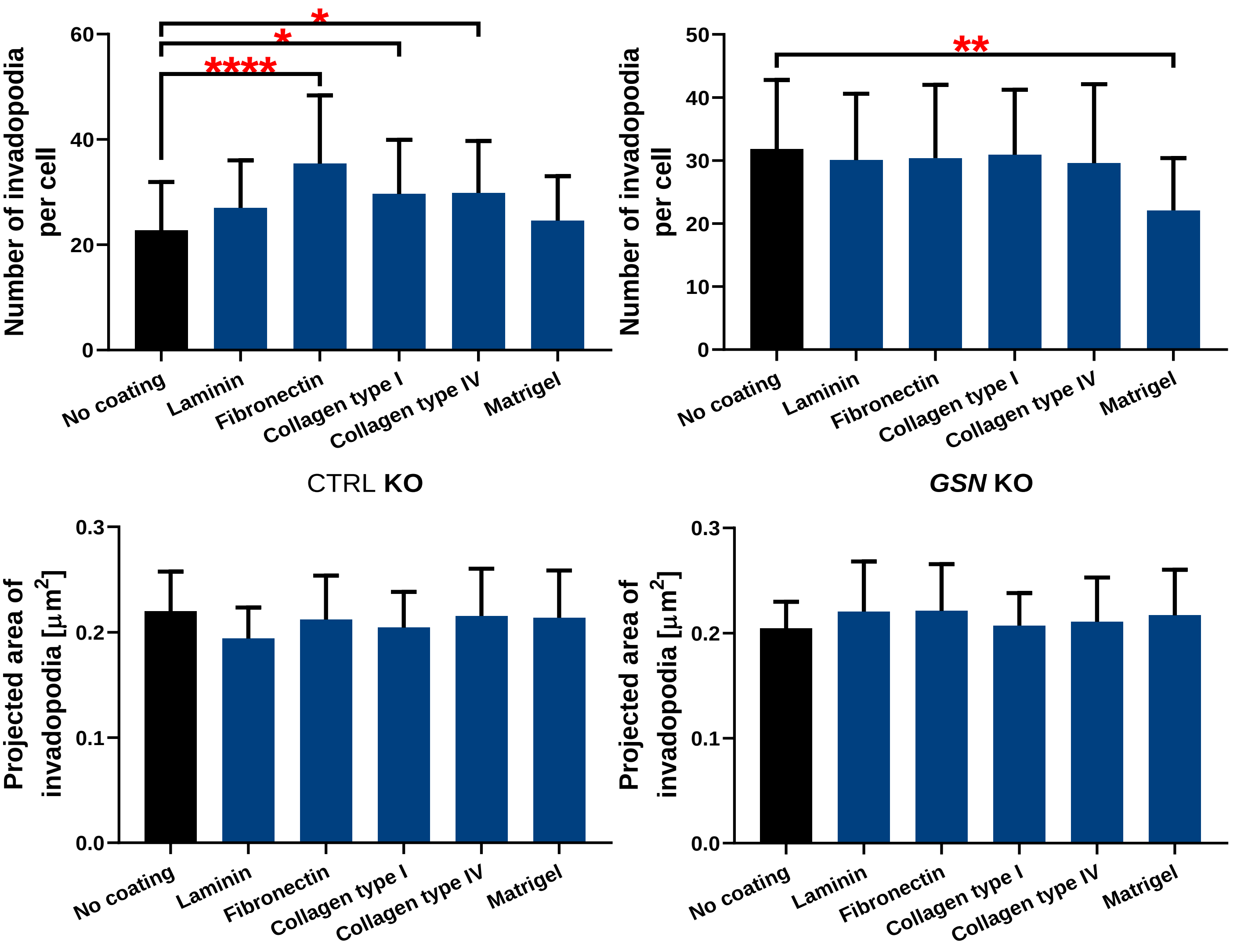

Fig. S9 Evaluation of the impact of different ECM proteins on the formation of filopodia, thick actin bundles and invadopodia in control and *GSN* KO clones. The cells were seeded onto ECM proteins and 48 h later fixed and stained to detect cortactin and F-actin. Microphotographs were taken with the help of SIM microscopy. Basing on the photos and methods described in the Materials and methods section the number, length and density of filopodia (**A**), the number of thick actin bundles (**B**), and the number of invadopodia (**C**) were measured ($n = 30$). Results are expressed as the mean ± SD; $p \le 0.05$ (*), $p \le 0.01$ (**), $p \le 0.001$ (***) and $p \le 0.0001$ (****). This figure corresponds to Fig. 7 in the main text. The results were calculated separately for the CTRL KO and *GSN* KO clones; ordinary and Kruskal-Wallis one-way ANOVA tests with post hoc (Dunn's multiple comparisons) test.

.

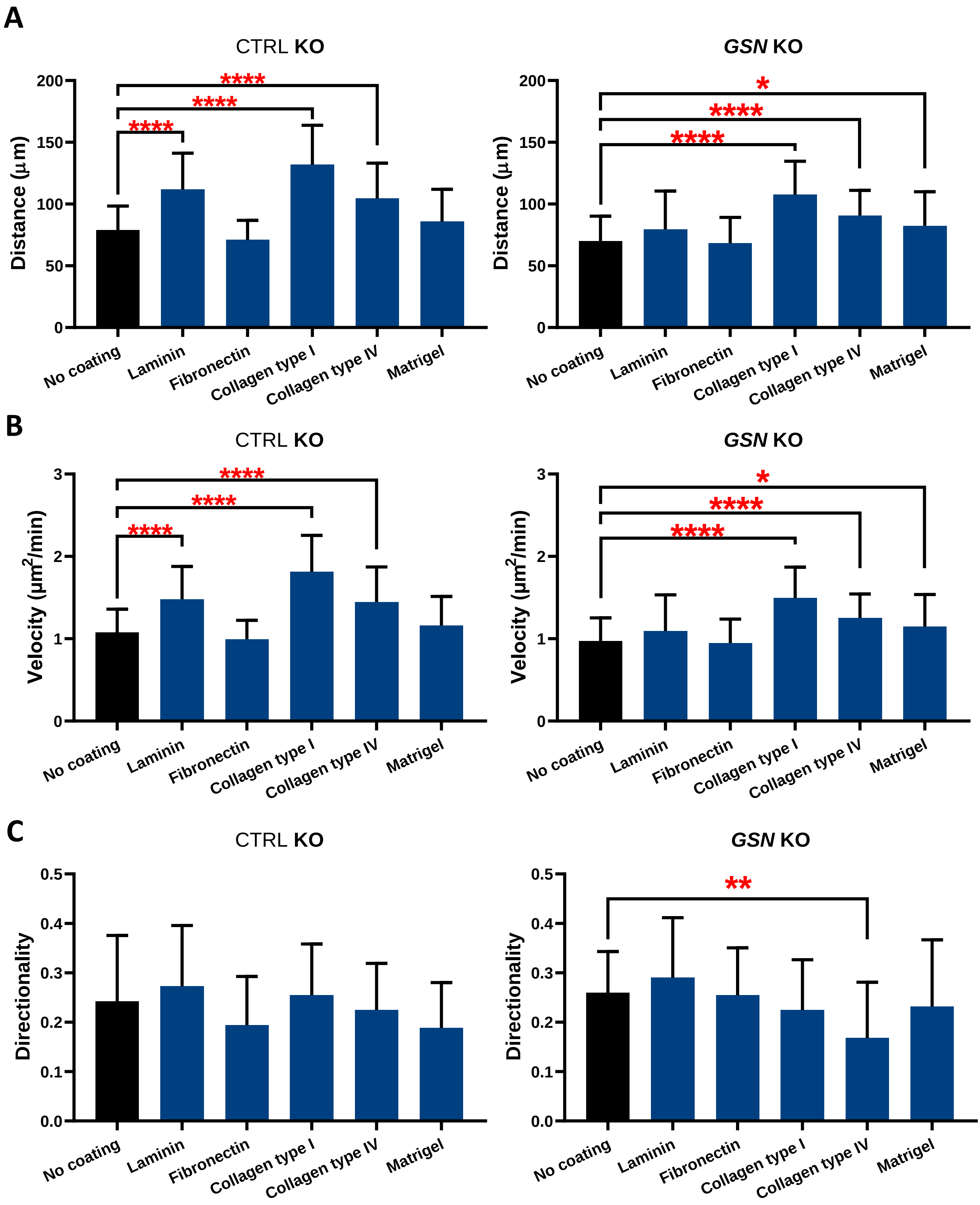

Fig. S10 ECM proteins differently influence spontaneous migration of control and *GSN* KO clones. The cells were seeded into coated wells with different ECM proteins of 96-well ImageLock plate. For 72 h hours the cells were monitored with the IncuCyte system. Next trajectories of single cells ($n = 90$) were plotted and (**A**) distance ($n = 90$), (**B**) velocity ($n = 90$) and (**C**) directionality ($n = 30$) of moving cells were assessed. Results are expressed as the mean ± SD; $p \leq 0.05$ (*), $p \leq 0.01$ (**) and $p \leq 0.0001$ (****). This figure corresponds to Fig. 8 in the main text. The results were calculated separately for the CTRL KO and *GSN* KO clones; ordinary and Kruskal-Wallis one-way ANOVA tests with post hoc (Dunn's multiple comparisons) test.

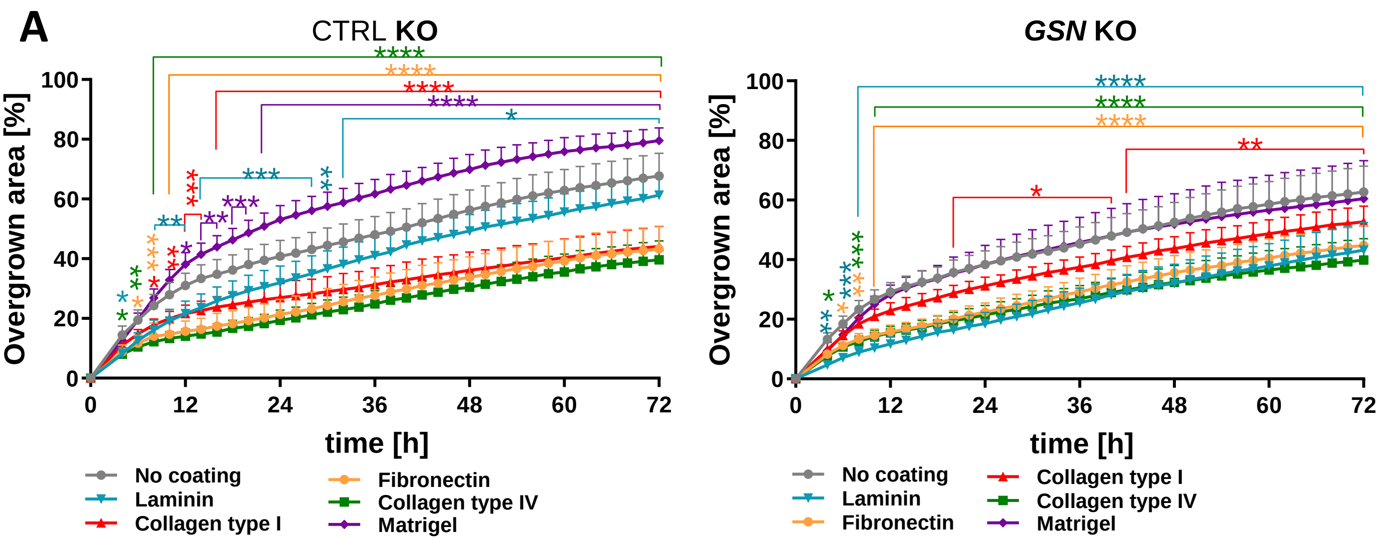

CTRL **KO**					
	Laminin	Fibronectin	Collagen I	Collagen IV	Matrigel
*	6 h	6 h	8 h	3 h	12 h
**	8-12 h, 30h	-	10 h	6 h	14-16 h
***	14-28 h	8 h	12-14 h	-	18-20 h
****	32-72 h	10-72 h	16-72 h	8-72 h	22-82 h

GSN **KO**					
	Laminin	Fibronectin	Collagen I	Collagen IV	Matrigel
*	-	6 h	20-40 h	6 h	-
**	4 h	8 h	42-72 h	-	-
***	6 h	-	-	8 h	-
****	8-72 h	10-72 h	-	10-72 h	-

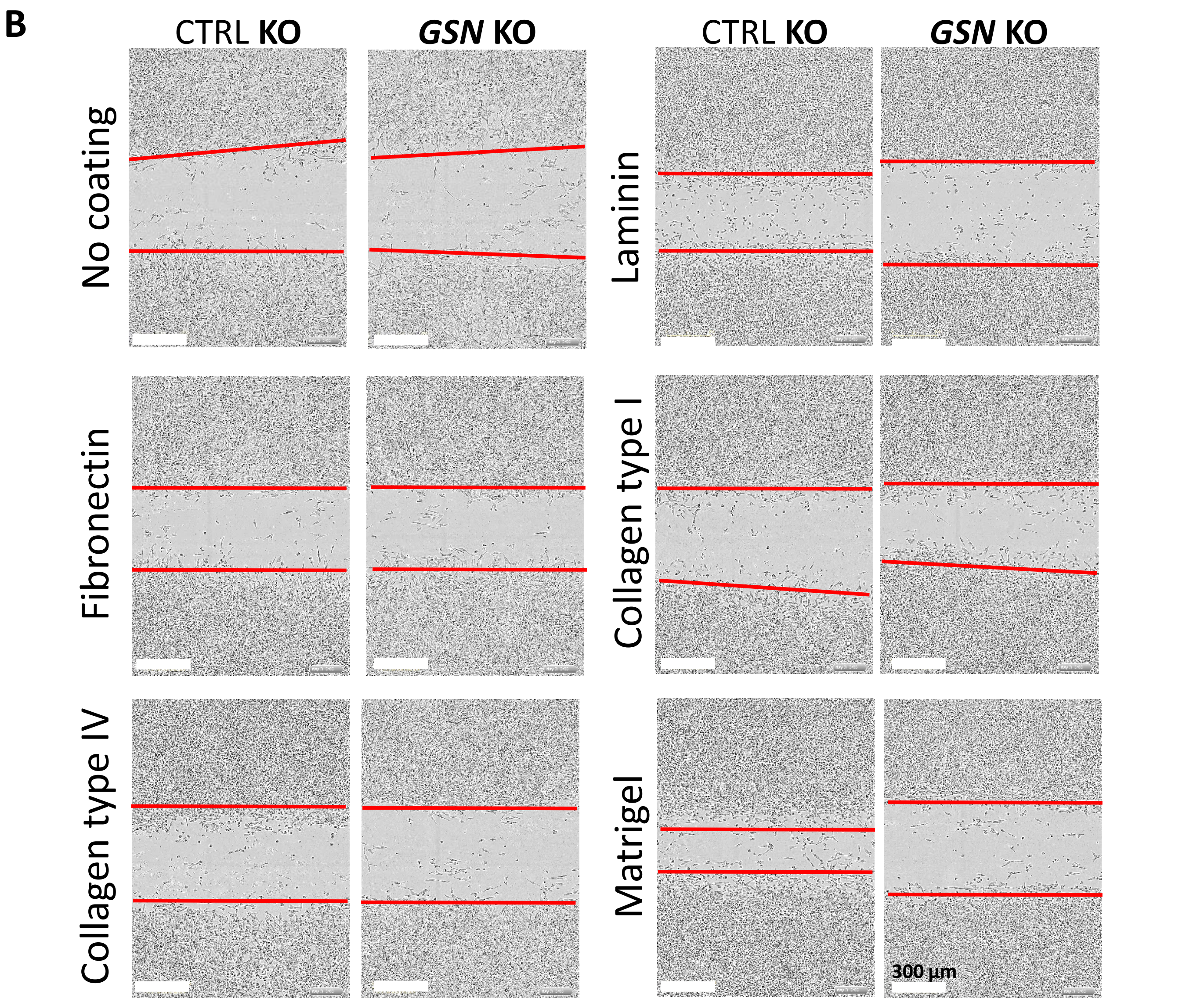

Fig. S11 Depending on the ECM protein there are differences in collective migration of control and *GSN* KO clones. (**A**) The cells upon making the wound with Wound Maker were monitored for 72 h. Every 2 h a photo was taken. Next the percentage of overgrown scratch by the cells over time was calculated with IncuCyte software ($n = 6$). Statistical significance is shown both on the graphs and in a table; $p \leq 0.01$ (*), $p \leq 0.01$ (**), $p \leq 0.001$ (***) and $p \leq 0.0001$ (****). The results were calculated separately for CTRL KO and *GSN* KO clones; two-way ANOVA with post hoc (Dunn's multiple comparisons) test (**B**) Representative photos taken 72 h after seeding the cells onto studied here ECM proteins. This figure corresponds to Fig. 9 in the main text.
